# Genetically mimicked effects of thyroid dysfunction on diabetic retinopathy risk: a 2-sample univariable and multivariable Mendelian randomization study

**DOI:** 10.3389/fendo.2024.1374254

**Published:** 2024-10-21

**Authors:** Junlin Ouyang, Ling Zhou, Qing Wang, Wei Yan

**Affiliations:** ^1^ China–Japan Union Hospital of Jilin University, Department of Endocrinology, Jilin, China; ^2^ China–Japan Union Hospital of Jilin University, Department of Obstetrics and Gynecology, Jilin, China; ^3^ China-Japan Union Hospital of Jilin University, Emergency Department, Jilin, China

**Keywords:** thyroid dysfunction, diabetic retinopathy, Mendelian randomization, meta-analysis, gene

## Abstract

**Background:**

Thyroid dysfunction exhibits a heightened prevalence among people with diabetes compared to those without diabetes. Furthermore, TD emerges as a notable correlated risk factor for the onset of diabetic retinopathy.

**Methods:**

Using data from the FinnGen database (R9), we investigated the causal relationship between thyroid dysfunction (TD) and four stages of diabetic retinopathy (DR). A two-sample univariable Mendelian randomization (UVMR) approach was employed to estimate the total causal effect of TD on four stages of DR, while multivariable Mendelian randomization (MVMR) was used to assess the direct causal effect. The meta-analysis was conducted to summarize the collective effect of TD on four stages of DR. The inverse variance weighted (IVW) method was the primary approach for Mendelian randomization analysis, with heterogeneity, horizontal pleiotropy, and leave-one-out sensitivity analyses performed to validate the robustness of the findings.

**Results:**

In UVMR analysis, thyrotoxicosis (TOS) was significantly associated with an increased risk of diabetic retinopathy across four stages (OR, 1.10–1.19; P<0.025). However, MVMR analysis, after adjusting for Graves’ disease (GD) and/or rheumatoid arthritis (RA), revealed no significant association between TOS and the four stages of diabetic retinopathy. The Meta-analysis demonstrated the collective effect of TOS on diabetic retinopathy across all stages [OR=1.11; 95% CI (1.08–1.15); P<0.01]. In UVMR analysis, the estimates for hypothyroidism (HPT) and GD were similar to those for TOS. In the MVMR analysis, after adjusting for RA, the significant effect of HPT on DR and non-proliferative diabetic retinopathy (NPDR) remained. Additionally, MVMR analysis suggested that the estimates for GD on DR were not affected by TOS, except for GD-proliferative diabetic retinopathy (PDR). However, no significant correlation persisted after adjusting for RA, including for GD-PDR.

**Conclusion:**

Our study demonstrated a significant association between thyroid dysfunction TD and DR, with the relationship being particularly pronounced in HPT-DR.

## 1 Introduction

Diabetic retinopathy(DR), a prominent microvascular complication of diabetes, stands as a leading cause of adult blindness (1). Recent investigations highlight a global prevalence of 34.6% for DR (2). The pathophysiological spectrum of DR encompasses two primary categories: proliferative DR(PDR) and nonproliferative DR(NPDR). In cases where PDR or macular edema with central involvement manifests, intravitreal anti-vascular endothelial growth factor(VEGF) therapy proves efficacious. However, the challenge of rapid recurrence upon drug discontinuation remains a noteworthy concern (3). Nevertheless, due to cost-effectiveness, it remains unrecognized for the treatment of NPDR. DR has garnered growing public concern owing to its widespread prevalence, expensive treatment modalities, and adverse impact on health. Consequently, it is a rising imperative to investigate etiological factors to avert the development of DR. Recent studies have reported that thyroid dysfunction(TD) may elevate the prevalence of DR (4).

TD, encompassing thyrotoxicosis(TOS), hypothyroidism(HPT), and autoimmune thyroid diseases. Diabetes mellitus(DM) and TD are prevalent endocrine system maladies in clinical practice, exhibiting an inherent connection (5). Blood glucose plays a pivotal role in regulating the hypothalamic-pituitary-thyroid axis and the release of thyroid-stimulating hormone(TSH) from the pituitary. Simultaneously, it influences the conversion of thyroxine to triiodothyronine in peripheral tissues (5). Concurrently, numerous observational studies highlight a higher prevalence of TD in diabetic populations compared to non-diabetic counterparts, particularly in type 1 diabetes mellitus, suggesting a robust shared genetic susceptibility. Serum levels of TSH represent an independent risk factor for DR (6). Despite this, the role of TD remains insufficiently emphasized in the context of DR prevention and treatment. Consequently, the paper aims to investigate the potential relationship between TD and diabetic retinopathy.

Nevertheless, existing evidence regarding the link between TD and diabetic retinopathy primarily stems from observational studies, introducing challenges such as confounding bias and reverse causality. To more accurately assess the potential causal relationships between TD, including TOS, HPT and Graves’ disease (GD), and various stages of DR, genetic approaches have surfaced as a reliable alternative for assessing causality. The approach helps circumvent interference from confounding or reverse causality (7). Mendelian randomization(MR) stands out as a robust analytical method for accomplishing this objective. Grounded in Mendel’s laws, MR is less susceptible to confounding factors. Its emphasis lies in investigating the causal relationship between the exposures and the outcomes through the utilization of genetic variants that meet three fundamental assumptions as instrumental variables(IVs) (8).

## 2 Materials and methods

### 2.1 Study design

**Figure 1:**
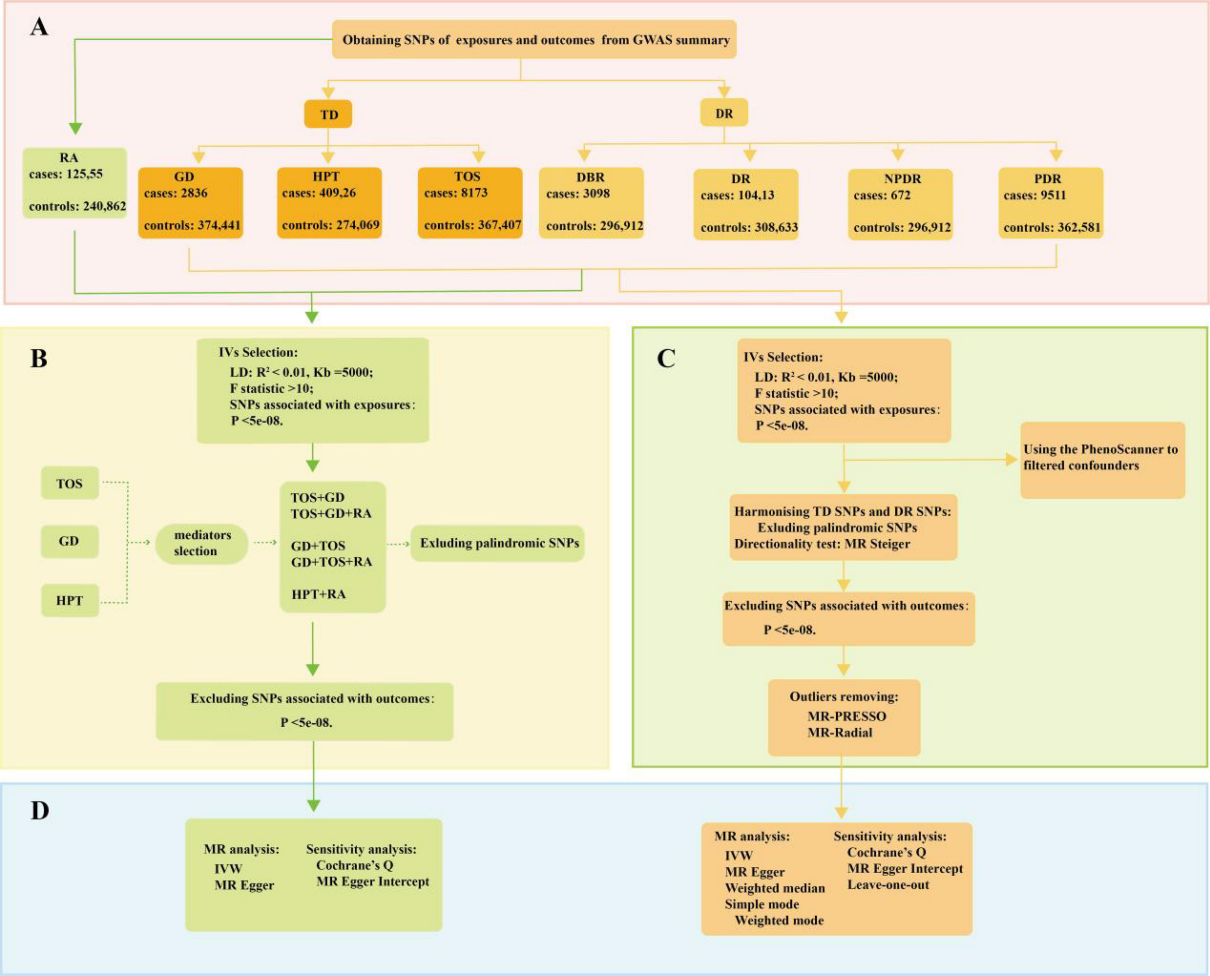
A flow diagram of the process in the MR analysis. **(A)** The SNPs data in the MR; **(B)** the selection of the IVs in the MVMR; **(C)** the selection of the IVs in the UVMR; **(D)** the analysis methods of MR.

As illustrated in **Figure 1**, the study design was based on the latest genome-wide association studies from the FinnGen database (R9) focusing on TD, rheumatoid arthritis (RA), and four stages of DR: background diabetic retinopathy (DBR), DR, NPDR, and PDR. Pooled data were analyzed using two-sample univariable Mendelian randomization (UVMR) to estimate the total causal effect of each exposure and multivariable Mendelian randomization (MVMR) to assess the direct causal effects of multiple exposures simultaneously. Meta-analysis was conducted for the pooled analysis. Additionally, a series of sensitivity analyses were performed to evaluate potential biases, including heterogeneity and horizontal pleiotropy, within the MR framework. The implementation of MR analysis relies on three critical assumptions, constituting an indispensable prerequisite for conducting analyses (9, 10): (1) Correlation: A robust correlation must exist between genetic variants and exposures; (2) Independence: Genetic variation should demonstrate independence from confounding factors; (3) Exclusion restriction: Genetic variation should exclusively influence the outcome through the targeted exposure factors. The present reporting and analysis procedures adhered to the STROBE-MR guidelines (11). **Table 1** provides a concise overview of the characteristics of the data sources utilized for the MR analysis, all of which are publicly accessible.

**Table 1:** Descriptive details of the sources of TD and DR.

Phenotype	TOS^Ia^	HPT^II^	GD^Ib^	RA^VII^	DR^III^	DBR^IV^	NPDR^V^	PDR^VI^
ncases	8173	40926	2836	12555	10413	3098	672	9511
Female gender (%)	79.0	80.0	84.5	67.3	43.9	45.8	41.1	47.9
Age at diagnosis (mean)	51.72	51.76	49.0	52.1	57.02	49.7	46.7	55.8
Data source	the FinnGen							

^I-VII^Data from the FinnGen all consist of European cohorts.

^Ia^GWAS ID: finngen_R9_THYROTOXICOSIS;

^II^GWAS ID: finngen_R9_E4_HYTHY_AI_STRICT;

^Ib^GWAS ID: finngen_R9_E4_GRAVES_STRICT;

^III^GWAS ID: finngen_R9_DM_RETINOPATHY_EXMORE;

^IV^GWAS ID: finngen_R7_DM_BCKGRND_RETINA;

^V^GWAS ID: finngen_R7_DM_BCKGRND_RETINA_NONPROLIF;

^VI^GWAS ID: finngen_R9_DM_RETINA_PROLIF;

^VII^GWAS ID: finngen_R9_M13_RHEUMA.

### 2.2 Instrumental variables

In the study, we queried the FinnGen(R9) database to identify key single-nucleotide polymorphisms(SNPs) serving as IVs, guided by three fundamental hypotheses (**Figure 1**). Firstly, we evaluated the correlation of IVs with exposures using genome-wide significance levels (P < 5e-08) and an F statistic (>10) (12). The F statistic was computed using the following formula:


$$\text{F}=\left({\text{R}}^{2}/\text{k}\right)/\left[\left(1-{\text{R}}^{2}\right)/\left(\text{n}-\text{k}-1\right)\right]$$

R^2^ is defined as the ability of the genetic variance to explain the exposure; k is the number of IVs used in the model; and n is the sample size.

Secondly, linkage disequilibrium (LD) was employed to guarantee the independence of IVs from other genes (kb = 5000, r^2^ = 0.01) (13, 14). Proxy SNPs were not used in cases where there were no SNPs associated with exposures in the outcomes. Additionally, we harmonized genetic variation by merging exposures and outcomes, concurrently eliminating palindromic SNPs. Lastly, SNPs significantly linked to outcomes (P < 5e-08) were also excluded.

### 2.3 Mendelian randomization

In the UVMR analysis, several MR methods were employed to substantiate total causal effect between TD and four stages of DR (**Figure 1**). These methods encompassed inverse variance weighting(IVW), MR-Egger, weighted median(WM), Simple mode, and Weighted mode (15). Among these, IVW, grounded on the equilibrium assumption of horizontal pleiotropy of IVs, stands out for its ability to disregard IVs’ heterogeneity, rendering it the most frequent approach in MR analysis (16). Other MR methods were employed to offer supplementary evaluations. The MR-Egger method, akin to IVW, differs primarily in considering the presence of intercept term in the regression. Furthermore, WM estimates furnish a reliable evaluation of causality even when only 50% of the IVs are valid (17). In MVMR analyses, the estimation of the direct causal effect of TD on four stages of DR predominantly relies on IVW and MR-Egger regression (**Figure 1**).

### 2.4 Sensitivity analyses

Subsequent to detecting causal effects through the aforementioned methods, we conducted sensitivity analyses to scrutinize the robustness of the MR findings, encompassing tests for heterogeneity and horizontal pleiotropy (**Figure 1**). In UVMR analyses, the identification of outliers is significantly reliant on the MR-PRESSO test and the RadialMR (9). Cochrane’s Q statistic (P > 0.05) and MR-Egger Intercept (P > 0.05) were separately employed in the study to assess the heterogeneity and horizontal pleiotropy (18). Furthermore, we evaluated the impact of individual SNP on the pooled causal estimates using the leave-one-out method, aiming to discern the presence of potentially pleiotropic SNPs that might influence the causal estimates (19). Statistical power for the UVMR study was calculated using the mRnd online tool (https://shiny.cnsgenomics.com/mRnd/). We employed the MR Steiger directionality test to evaluate the directionality of UVMR causal estimates. The TwoSampleMR and MendelianRandomization packages were utilized to identify potential confounders. In MVMR analyses, Cochrane’s Q statistic was primarily used to assess study heterogeneity, and MR-Egger intercept was employed to evaluate horizontal pleiotropy.

### 2.5 Statistical analyses

The TwoSampleMR package and the RadialMR package were employed for UVMR analyses within the R (version 4.2.3) (20, 21). For MVMR analyses, we utilized the MendelianRandomization package. Meta-analyses were conducted using the Meta package. A significance threshold of P < 0.05 was established for statistical significance. Bonferroni correction was applied to redefine the threshold of statistical significance (P < 0.05/n) to account for multiple testing, where n denotes the number of MR tests (14). The adjusted p-value for the TD-diabetic retinopathy (DBR/DR/NPDR/PDR) analysis was set at 0.025. Similarly, the MVMR analyses also employed an adjusted p-value (0.025). The OR, beta values and their respective 95% confidence intervals were utilized to furnish estimates of relative risk.

## 3 Results

The UVMR study examined the total causal effect between TD and DR using three different exposures and four stages of outcomes. All IVs were significantly associated with the exposures (P<5e-08) and had F-statistics greater than 10 (**Supplementary Tables 1**–**12**), indicating a strong association with the exposure. Additionally, outliers were eliminated by MR-PRESSO (**Supplementary Table**: MR results sum1-12) and the RadialMR (**Supplementary Figures 1**–**3**), and each SNP was not associated with the outcome (P>5e-08) (**Supplementary Tables 1**–**12**). Given GD is a known etiology of TOS, the pleiotropic effects of TOS and GD may violate the exclusion restriction hypothesis. Our analysis using the Phenoscanner package identified RA as a potentially influential confounder or mediator (**Supplementary Table**: potential confounders), with SNPs associated with RA detailed in **Supplementary Table 13**. Furthermore, prior reports have hinted at ambiguous associations of RA with both diabetic retinopathy and TD (22–24). Consequently, we employed MVMR to adjust for TOS, GD, and RA.

### 3.1 Sensitivity analyses

The results of the UVMR analyses passed Cochrane’s Q test (**Tables 2**, **3**). With the exception of the GD-NPDR analysis, all UVMR analyses passed the MR-Egger intercept test (**Tables 2**, **3**). Leave-one-out analyses confirmed that the causality observed in the UVMR analyses was not driven by any single SNP (**Supplementary Figures 4**–**6**). Additionally, the MR Steiger directionality test results supported the accuracy of our causal direction estimates (**Supplementary Tables 1**–**12**), further validating the robustness of the UVMR findings. The statistical power of the UVMR studies, as calculated using the web tool, was all greater than 0.9. Although the heterogeneity test (P<0.05) indicated some heterogeneity in the MVMR analyses, no horizontal pleiotropy was detected (P>0.05). The meta-analysis showed heterogeneity in both the overall and partial subgroup analyses, therefore, the random-effects model was used to combine the effect sizes.

**Table 2:** MR results of TOS on DBR/DR/NPDR/PDR.

Exposures	Outcomes	Method	NSNP	OR	95%CI	P value	Cochrane’s Q	MR–Egger test	
							p-value	Intercept	p-value
TOS	DBR	MR Egger	32	1.39	1.06-1.82	0.03	0.10	-0.02	0.27
		WM	32	1.09	0.96-1.22	0.18			
		IVW	32	1.19	1.09-1.31	0.00009	0.09		
	DR	MR Egger	27	1.19	1.00-1.40	0.05	0.11	-0.01	0.34
		WM	27	1.07	1.00-1.15	0.05			
		IVW	27	1.10	1.04-1.16	0.001	0.11		
	NPDR	MR Egger	37	1.58	1.04-2.42	0.0396	0.52	-0.05	0.16
		WM	37	1.20	0.96-1.50	0.0933			
		IVW	37	1.19	1.03-1.37	0.0218	0.47		
	PDR	MR Egger	32	1.10	0.97-1.25	0.14	0.64	0.00	0.94
		WM	32	1.06	1.00-1.13	0.06			
		IVW	32	1.10	1.05-1.15	2.8E-05	0.69		

**Table 3:** MR results of HPT on DBR/DR/NPDR/PDR.

Exposures	Outcomes	Method	NSNP	OR	95%CI	P value	Cochrane’s Q	MR–Egger test	
							p-value	Intercept	p-value
HPT	DBR	MR Egger	152	1.49	1.21-1.84	0.0002	0.73	-0.0026	0.73
		WM	152	1.38	1.23-1.54	1.8E-08			
		IVW	152	1.44	1.34-1.55	8.4E-24	0.75		
	DR	MR Egger	144	1.09	0.97-1.23	0.16	0.63	0.01	0.14
		WM	144	1.14	1.07-1.22	4.2E-05			
		IVW	144	1.19	1.14-1.24	6.4E-16	0.61		
	NPDR	MR Egger	179	2.22	1.50-3.29	9.0E-05	0.83	-0.02	0.09
		WM	179	1.58	1.29-1.94	1.2E-05			
		IVW	179	1.62	1.42-1.86	3.1E-12	0.80		
	PDR	MR Egger	175	1.10	0.99-1.23	0.09	0.41	0.00	0.42
		WM	175	1.13	1.07-1.21	4.5E-05			
		IVW	175	1.15	1.11-1.19	7.6E-13	0.42		

### 3.2 Univariable Mendelian randomization

In populations of European ancestry (EA), IVW analyses indicated a potential causal relationship between genetically predicted TOS and DBR/DR/NPDR/PDR (**Table 2**). However, WM and MR-Egger did not provide similar evidence in the TOS-DBR/DR/NPDR/PDR analyses (P>0.025). Notably, the OR values from both WM and MR-Egger were all greater than 1 (**Table 2**), and the scatter plot suggested a positive correlation between TOS and DBR/DR/NPDR/PDR (**Supplementary Figure 7**), indicating consistency in the directionality of the results. Sensitivity analyses showed no evidence of bias, supporting the validity of the findings. Similarly, in the HPT-DR analyses, IVW indicated that genetically predicted HPT was potentially causally related to DBR/DR/NPDR/PDR (**Table 3**). In the MR results for HPT-DR/PDR, MR-Egger did not provide supporting evidence (P>0.025). However, WM provided consistent estimates, and the ORs from MR-Egger and WM were all greater than 1 (**Table 3**). The scatter plot further demonstrated a potential positive correlation between HPT and DR/PDR (**Supplementary Figures 8B, D**), which aligns with our overall interpretation of the MR results.

In contrast to the previous findings, IVW, WM, and MR-Egger all indicated a potential causal relationship between genetically predicted GD and DR/DBR/NPDR/PDR (**Table 4**).

**Table 4:** MR results of GD on DBR/DR/NPDR/PDR.

Exposures	Outcomes	Method	NSNP	OR	95%CI	P value	Cochrane’s Q	MR–Egger test	
							p-value	Intercept	p-value
GD	DBR	MR Egger	11	1.59	1.3-1.94	0.001	0.52	-0.02	0.51
		WM	11	1.48	1.32-1.66	3.7E-11			
		IVW	11	1.49	1.38-1.62	2.7E-23	0.57		
	DR	MR Egger	11	1.29	1.16-1.44	0.001	0.79	-0.01	0.66
		WM	11	1.26	1.19-1.34	1.5E-13			
		IVW	11	1.26	1.20-1.32	1.2E-24	0.84		
	NPDR	MR Egger	16	2.47	1.77-3.46	0.0001	0.60	-0.15	0.01
		WM	16	1.51	1.23-1.84	5.6E-05			
		IVW	16	1.50	1.28-1.76	4.2E-07	0.11		
	PDR	MR Egger	17	1.24	1.11-1.38	0.0013	0.11	-0.02	0.19
		WM	17	1.17	1.11-1.24	6.4E-09			
		IVW	17	1.16	1.11-1.21	1.1E-10	0.07		

### 3.3 Multivariable Mendelian randomization

When accounting for GD and/or RA, both IVW and MR-Egger analyses suggested that genetically predicted TOS did not significantly increase the risk of DBR/DR/NPDR/PDR, though the results were not statistically significant (**Tables 5**, **6**).

**Table 5:** MVMR results (IVW) of TD on DBR/DR/NPDR/PDR.

Items	Model 1			Model 2		
	β	95%CI	P-value	β	95%CI	P-value
TOS						
DBR	-0.56	-1.24, 0.12	0.104	-0.05	-0.71, 0.61	0.873
DR	-0.31	-0.74, 0.12	0.153	-0.05	-0.48, 0.39	0.836
NPDR	-0.54	-1.32, 0.23	0.17	-0.02	-0.71, 0.68	0.96
PDR	-0.10	-0.38, 0.19	0.515	0.06	-0.24, 0.37	0.681
HPT						
DBR	0.16	-0.02, 0.35	0.088	–	–	–
DR	0.18	0.06, 0.30	0.005	–	–	–
NPDR	0.29	0.07, 0.52	0.012	–	–	–
PDR	0.09	-0.001, 0.18	0.052	–	–	–
GD						
DBR	0.79	0.27, 1.30	0.003	0.23	-0.31, 0.76	0.406
DR	0.42	0.10, 0.74	0.011	0.13	-0.23, 0.48	0.476
NPDR	0.85	0.26, 1.44	0.005	0.23	-0.33, 0.79	0.412
PDR	0.21	-0.01, 0.42	0.062	0.02	-0.23, 0.26	0.887

Model 1^TOS^ adjusted for: GD; Model 1^HPT^ adjusted for: RA; Model 1^GD^ adjusted for: TOS. Model 2^TOS^ adjusted for: GD and RA; Model 2^GD^ adjusted for: TOS and RA.

**Table 6:** MVMR results (MR-egger) of TD on DR (DBR/DR/NPDR/PDR).

Items	Model 1			Model 2		
	β	95%CI	P-value	β	95%CI	P-value
TOS						
DBR	-0.58	-1.51, 0.36	0.229	0.14	-0.62, 0.89	0.718
DR	-0.27	-0.85, 0.31	0.362	0.06	-0.44, 0.57	0.810
NPDR	-0.11	-1.18, 0.96	0.838	0.19	-0.62, 0.99	0.646
PDR	-0.02	-0.40, 0.37	0.931	0.18	-0.17, 0.53	0.318
HPT						
DBR	0.35	-0.05, 0.74	0.087	–	–	–
DR	0.51	0.23, 0.78	0.000	–	–	–
NPDR	0.56	0.08, 1.03	0.022	–	–	–
PDR	0.31	0.12, 0.50	0.002	–	–	–
GD						
DBR	0.79	0.27, 1.31	0.003	0.27	-0.27, 0.81	0.329
DR	0.42	0.09, 0.74	0.012	0.15	-0.21, 0.51	0.408
NPDR	0.87	0.28, 1.45	0.004	0.27	-0.29, 0.84	0.343
PDR	0.21	-0.01, 0.42	0.063	0.04	-0.21, 0.29	0.747

Model 1^TOS^ adjusted for: GD; Model 1^HPT^ adjusted for: RA; Model 1^GD^ adjusted for: TOS. Model 2^TOS^ adjusted for: GD and RA; Model 2^GD^ adjusted for: TOS and RA.

When RA was considered, both IVW and MR-Egger analyses suggested that genetically predicted HPT could still increase the risk of developing DR (DR/NPDR) (**Tables 5**, **6**). However, for DBR, genetically predicted HPT was found to be non-significant after adjusting for RA in both IVW and MR-Egger analyses (**Tables 5**, **6**). While IVW analysis indicated that genetically predicted HPT was not significantly associated with PDR after adjusting for RA (**Table 5**), MR-Egger analysis suggested that genetically predicted HPT could still increase the risk of PDR (**Table 6**), with both MR-Egger and IVW analyses showing a positive association. Therefore, the study concludes that HPT may still increase the risk of PDR after adjusting for RA.

When considering TOS, both IVW and MR-Egger analyses indicated that genetically predicted GD could still increase the risk of developing DBR/DR/NPDR (**Tables 5**, **6**). However, when both TOS and RA were considered, genetically predicted GD was no longer associated with DBR/DR/NPDR/PDR (**Tables 5**, **6**).

### 3.4 Meta-analysis

**Figure 2:**
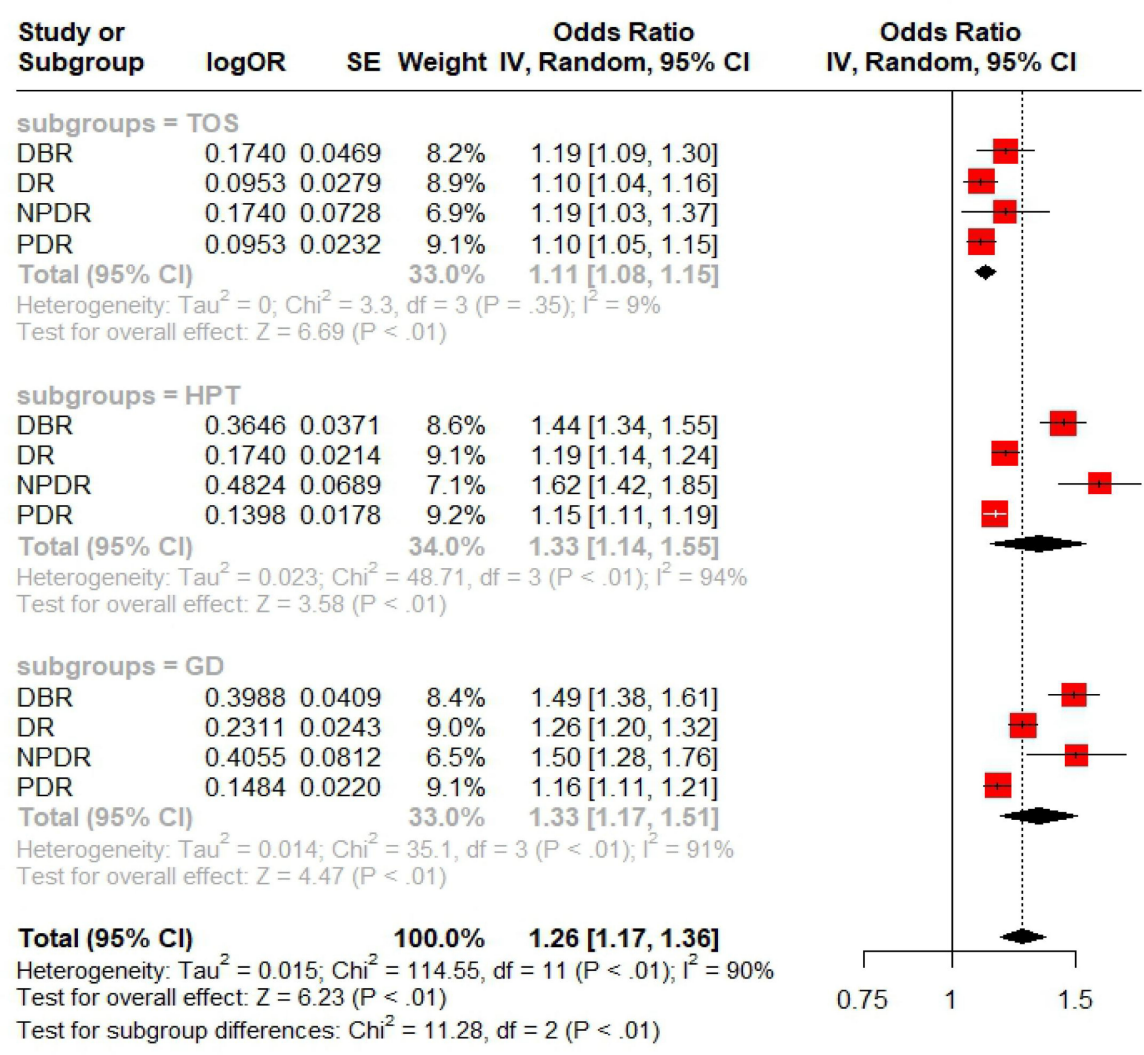
Forest plots of Meta analysis on TD-DBR/DR/NPDR/PDR.

In UVMR analysis, this study assessed the effect of TD on diabetic retinopathy across its four stages. A meta-analysis, conducted using R software, summarized the overall impact of TD on DBR/DR/NPDR/PDR based on UVMR results. As shown in **Figure 2**, the effect of GD and HPT on DBR/DR/NPDR/PDR was greater than that of TOS, with the difference being statistically significant (P<0.01). Although the meta-analysis showed significance in both subgroup and overall analyses (P<0.01), substantial heterogeneity was noted in the HPT/GD subgroup as well as in the overall estimates.

## 4 Discussion

The two-sample UVMR study demonstrated that genetically predicted TD, particularly HPT, can increase the risk of diabetic retinopathy in patients with DM, with the robustness of the analytical procedure confirmed. In MVMR analysis, no significant association was found between TOS and diabetic retinopathy after adjusting for GD. However, GD remained significantly associated with diabetic retinopathy, except for GD-PDR, after adjusting for TOS. This observed difference is thought-provoking. The study by Lin D et al. (6) provides a plausible explanation for the discrepancy. They found that bovine TSH (b-TSH) promotes glucose-induced mitochondrial apoptosis in human peripapillary retinal cells, whereas blocking TSHR significantly inhibits mitochondrial apoptosis in a high-glucose environment (6). In thyrotoxicosis, elevated levels of thyroxine and triiodothyronine inhibit TSH production via negative feedback on the hypothalamic-pituitary-thyroid axis. In contrast, in GD, thyroid-stimulating antibodies (TSAb) mimic the effects of TSH by binding to TSHR, which may explain the observed difference. In contrast, hypothyroidism involves positive feedback activation of the hypothalamic-pituitary-thyroid axis. Both univariate and multivariate MR analyses indicated a significant association between HPT and diabetic retinopathy, aligning with a prior meta-analysis of observational studies that reported a significant correlation between hypothyroidism and diabetic retinopathy (OR=2.13, 95% CI=1.41–3.23, P<0.001) (25). A subsequent meta-analysis reached a similar conclusion (26). Additionally, a case-control study demonstrated a significant association between hypothyroidism and an increased prevalence of diabetic retinopathy in DM patients, even after adjusting for age, gender, diabetes duration, glycosylated hemoglobin, BMI, hypertension, and LDL cholesterol (27). Furthermore, higher thyrotropic hormone levels have been linked to narrower retinal arterioles and lower arteriovenous indices in patients with hypothyroidism compared to those with normal thyroid function (28). In the pooled analysis, we observed that the overall effect of HPT on diabetic retinopathy was greater than that of TOS or GD. While our findings, along with previous studies, seem to implicate TSH as the key factor driving the increased risk of diabetic retinopathy associated with TD. But is this really the case? A retrospective study in a Caucasian population, for instance, found no significant association between TSH levels or hypothyroidism and diabetic retinopathy (29). Moreover, UVMR analysis suggested the presence of horizontal pleiotropy in GD-NPDR, but after adjusting for TOS, the pleiotropy disappeared, and both MR analyses indicated that GD could increase the risk of developing NPDR. This result seems to imply that thyrotoxicosis alone plays a role in the GD-NPDR axis. However, an MR study on thyroid hormones and microvascular complications in diabetes suggested that elevated thyroid hormone levels do not increase the risk of developing DR (30). This may be due to the fact that the study, when selecting IVs, focused solely on hormone levels and overlooked the disease itself. Additionally, variations in the sources of exposure-related IVs may have contributed to the differences in the analytical results.

Diabetic retinopathy constitutes a significant microvascular complication of diabetes (1), wherein microvascular injury plays a pivotal role in its pathogenesis. Various factors contribute to microvascular injury, including hypoxia, endothelial damage, oxidative stress, inflammatory response, and fibrovascular proliferation. In individuals with TD, both TOS and HPT are associated with elevated serum C-reactive protein levels, indicating a heightened systemic inflammatory response (5). Furthermore, conditions such as GD and Hashimoto thyroiditis can all result in increased serum VEGF levels (31). Individuals with HPT exhibit increased activity of plasma malondialdehyde, a specific indicator of oxidative stress levels (5). Moreover, thyroid hormones play a significant role in the normal development of retinal cellular structures, as evidenced by researchers discovering low levels of sirtuin2 in the retinal ganglion cell layer of hypothyroid mice (32). Therefore, the impact of TD on diabetic retinopathy may not solely be attributed to the level of TSH and thyroid hormone but rather the combined influence of numerous factors.

Compared to observational studies, the MR study can significantly mitigate confounding effects. However, the current MR design possesses both its own strengths and inherent limitations, primarily stemming from three essential assumptions that must be satisfied. Firstly, the correlation assumption was supported by the genome-wide significance level (P<5e-08) in the GWAS. Additionally, the UVMR studies are less susceptible to weak instrument bias, as we exclusively incorporated SNPs with substantial instrumental strength (F>10) while excluding those in linkage disequilibrium. Nevertheless, MVMR analyses exclusively considered SNPs with genome-wide significance levels (P<5e-08) and eliminated those in linkage disequilibrium. Secondly, the MR-Egger Intercept evaluated the horizontal pleiotropy in the UVMR analysis, indicating an absence of horizontal pleiotropy. However, during confounder screening, we identified RA as potentially influencing the causal chain of TD on diabetic retinopathy. Consequently, confounding was addressed through the MVMR analysis. Nevertheless, violations may persist, and alternative pleiotropic pathways from IVs to diabetic retinopathy remain unexplored in this study, necessitating investigation in future research. Thirdly, the genetic variant data predominantly relied on GWAS from European ancestry. The approach has the drawback of lacking the ability to fully represent the entire population. However, it offers the advantage of minimizing the risk of population-based confounding. Additionally, heterogeneity was observed in the MVMR analysis, but we employed the IVW with a random-effects model to evaluate the MVMR results. Lastly, given the presence of heterogeneity, the meta-analysis utilized a random-effects model to amalgamate effect sizes.

## 5 Conclusion

In conclusion, our study demonstrated a significant correlation between TD and diabetic retinopathy, with a particularly strong association for HPT. In the HPT-DBR/DR/NPDR/PDR MVMR analysis, HPT remained significantly associated with DBR/DR/NPDR/PDR even after adjusting for RA, suggesting that the impact of HPT on diabetic retinopathy is independent of RA.

## Data Availability

The original contributions presented in the study are publicly available. This data can be found here: https://github.com/ling-98/TD-DR-MR-analyses.git.
