# Supplementary material for: Genetically mimicked effects of thyroid dysfunction on diabetic retinopathy risk: a 2-sample univariable and multivariable Mendelian randomization study
Source: Front Endocrinol (Lausanne). 2024 Oct 21;15:1374254. doi: 10.3389/fendo.2024.1374254 (PMC11532173; doi:10.3389/fendo.2024.1374254)
Supplement: Supplementary file 1 [file DataSheet1.pdf]

STROBE-MR checklist of recommended items to address in reports of Mendelian randomization studies[1 2]

Item No.	Section		Checklist item	Page No.	Relevant text from manuscript
1	**TITLE and ABSTRACT**		Indicate Mendelian randomization (MR) as the study's design in the title and/or the abstract if that is a main purpose of the study		Genetically mimicked effects of thyroid dysfunction on diabetic retinopathy risk: a 2-sample univariable and multivariable Mendelian randomization study
	**INTRODUCTION**				
2	**Background**		Explain the scientific background and rationale for the reported study. What is the exposure? Is a potential causal relationship between exposure and outcome plausible? Justify why MR is a helpful method to address the study question		Thyroid dysfunction exhibits a heightened prevalence among people with diabetes compared to those without diabetes. Furthermore, Serum levels of TSH represent an independent risk factor for DR. TD emerges as a notable correlated risk factor for the onset of diabetic retinopathy.<br>Nevertheless, existing evidence regarding the link between TD and DR primarily stems from observational studies, introducing challenges such as confounding bias and reverse causality. The MR study has surfaced as a reliable alternative for assessing causality.
3	**Objectives**		State specific objectives clearly, including pre-specified causal hypotheses (if any). State that MR is a method that, under specific assumptions, intends to estimate causal effects		the paper aims to investigate the potential relationship between TD and DR.
	**METHODS**				
4	**Study design and data sources**		Present key elements of the study design early in the article. Consider including a table listing sources of data for all phases of the study. For each data source contributing to the analysis, describe the following:		As depicted in Figure 1 and table 1, the study design encompassed two-sample UVMR and MVMR analyses, utilizing summary statistics derived from the latest GWAS on TD, RA, and diabetic retinopathy from the FinnGen database.
		a)	Setting: Describe the study design and the underlying population, if possible. Describe the setting, locations, and relevant dates, including periods of recruitment, exposure, follow-up, and data collection, when available.		Genome-wide association studies (GWAS) from the FinnGen was used. Sample size, sex ratio, average age, subjects, etc are showed in table 1 and figure 1.
		b)	Participants: Give the eligibility criteria, and the sources and methods of selection of participants. Report the sample size, and whether any power or sample size		Genome-wide association studies (GWAS) from the FinnGen was used. Sample size, sex ratio, average age, subjects, etc are showed in table 1

			calculations were carried out prior to the main analysis	and figure 1.characterizes the unselected population.
		c)	Describe measurement, quality control and selection of genetic variants	1. SNPs associated with exposures:$P<5e-08$<br>2. LD: R2 < 0.01, Kb =5000;<br>3. F statistic >10;<br>4. Excluding SNPs associated with outcomes:$P<5e-08$.<br>Figure 1 provides more specific and detail process.
		d)	For each exposure, outcome, and other relevant variables, describe methods of assessment and diagnostic criteria for diseases	The definitions of each exposures and outcomes are described in the paper.
		e)	Provide details of ethics committee approval and participant informed consent, if relevant	
5	**Assumptions**		Explicitly state the three core IV assumptions for the main analysis (relevance, independence and exclusion restriction) as well assumptions for any additional or sensitivity analysis	1. Three key assumptions:<br>(1) Correlation: A robust correlation must exist between genetic variants and exposures.<br>(2) Independence: Genetic variation should demonstrate independence from confounding factors.<br>(3) Exclusion restriction: Genetic variation should exclusively influence the outcome through the targeted exposure factors.<br>2. Additionally, using a web tool (https://shiny.cnsgenomics.com), we calculated that the statistical power of the UVMR studies.<br>3. Bonferroni correction was applied to redefine the threshold of statistical significance (P < 0.05/n) to account for multiple testing.
6	**Statistical methods: main analysis**		Describe statistical methods and statistics used	
		a)	Describe how quantitative variables were handled in the analyses (i.e., scale, units, model)	We use the random-effect model to access these MR results. The statistical effect size or unit of measurement regarding exposures, outcomes, and covariables, therefore, we didn't report them.
		b)	Describe how genetic variants were handled in the analyses and, if applicable, how	Figure1 describes specific screening methods for genetic instrumental variables, and supplementary

			their weights were selected	tables provide details of the number of SNPs extracted.
		c)	Describe the MR estimator (e.g. two-stage least squares, Wald ratio) and related statistics. Detail the included covariates and, in case of two-sample MR, whether the same covariate set was used for adjustment in the two samples	IVW is the primary method of the MR study. We conducted a search for exposure-related covariates by R（TwoSampleMR and MendelianRandomization）. And we conducted the MVMR by adjusting RA, GD and TOS. The text already have specifically described these methods.
		d)	Explain how missing data were addressed	There were no missing data in this MR analysis
		e)	If applicable, indicate how multiple testing was addressed	We employed the Bonferroni correction, which adjusted the significance threshold to P<0.05/n, where 'n' represents the number of MR tests conducted
7	**Assessment of assumptions**		Describe any methods or prior knowledge used to assess the assumptions or justify their validity	The strength of instrumental variables was assessed using the F-statistic
8	**Sensitivity analyses and additional analyses**		Describe any sensitivity analyses or additional analyses performed (e.g. comparison of effect estimates from different approaches, independent replication, bias analytic techniques, validation of instruments, simulations)	Before conducting MR analysis, we employed the MR-PRESSO and RadiaMR to identify and remove outliers. Cochrane's Q and MR-Egger Intercept were employed to evaluate heterogeneity and horizontal pleiotropy among IVs. When the MR-Egger Intercept was significantly different from 0 (P < 0.05), we concluded the MVMR to adjust the horizontal pleiotropy. Using a web tool (https://shiny.cnsgenomics.com), we calculated that the statistical power.
9	**Software and pre-registration**			
		a)	Name statistical software and package(s), including version and settings used	The TwoSampleMR package and the RadialMR package were used to perform the UVMR analysis in the R(version 4.2.3). MendelianRandomization package was employed to conduct the MVMR. Meta-analyses were conducted using the Meta package.
		b)	State whether the study protocol and details were pre-registered (as well as when and where)	No

RESULTS

10	**Descriptive data**			
		a)	Report the numbers of individuals at each stage of included studies and reasons for exclusion. Consider use of a flow diagram	We already have provided the detail characteristic of demography in the part of methods. Figure 1 is process diagram. The paper didn't provide the information of excluded population. But we provided the excluded SNPs in the supplement tables.
		b)	Report summary statistics for phenotypic exposure(s), outcome(s), and other relevant variables (e.g. means, SDs, proportions)	The supplement tables provided these detail information.
		c)	If the data sources include meta-analyses of previous studies, provide the assessments of heterogeneity across these studies	The data sources do not include meta-analyses of previous studies.
		d)	For two-sample MR:<br>i. Provide justification of the similarity of the genetic variant-exposure associations between the exposure and outcome samples<br>ii. Provide information on the number of individuals who overlap between the exposure and outcome studies	The paper is tow-sample MR study.<br>1. All sample populations were from Europe, minimizing the risk of population-based confounding.<br>2. The justification is showed in supplement tables.<br>3. we lacked the means to precisely quantify participant overlap across the datasets utilized in this study.
11	**Main results**			
		a)	Report the associations between genetic variant and exposure, and between genetic variant and outcome, preferably on an interpretable scale	It is showed in Supplementary tables, including number of SNPs, sample size, confounding, and correlation of exposure with SNPs.
		b)	Report MR estimates of the relationship between exposure and outcome, and the measures of uncertainty from the MR analysis, on an interpretable scale, such as odds ratio or relative risk per SD difference	Odds ratios (OR), beta, and their corresponding 95% confidence intervals (CI) were utilized to estimate relative risk within our MR analysis.
		c)	If relevant, consider translating estimates of relative risk into absolute risk for a meaningful time period	No
		d)	Consider plots to visualize results (e.g. forest plot, scatterplot of associations between genetic variants and outcome versus between genetic variants and exposure)	Supplementary figures
12	**Assessment of assumptions**			

		a)	Report the assessment of the validity of the assumptions	The paper provided the assessment. Supplement tables provided the detail information.
		b)	Report any additional statistics (e.g., assessments of heterogeneity across genetic variants, such as I^2, Q statistic or E-value)	Supplementary tables provided Q statistic and P-value.
13	**Sensitivity analyses and additional analyses**			
		a)	Report any sensitivity analyses to assess the robustness of the main results to violations of the assumptions	inverse variance-weighted method (IVW), MR-Egger regression, weighted median (WM), simple mode, and weighted mode, MR-PRESSO, MR-radial. Their results were showed in Supplementary tables
		b)	Report results from other sensitivity analyses or additional analyses	Supplement figures provided leave-one-out method.
		c)	Report any assessment of direction of causal relationship (e.g., bidirectional MR)	No
		d)	When relevant, report and compare with estimates from non-MR analyses	We discussed the MR results and the results of clinical studies in the Discussion part.
		e)	Consider additional plots to visualize results (e.g., leave-one-out analyses)	Supplementary figures
	**DISCUSSION**			
14	**Key results**		Summarize key results with reference to study objectives	In the UVMR analysis, TOS exhibited a notable association with an elevated risk of diabetic retinopathy [OR = 1.10-1.19; P < 0.025]. However, the MVMR analysis, adjusted for GD and/or RA, indicated that TOS was not significantly associated with diabetic retinopathy. The meta-analysis provided a collective effect of TOS on diabetic retinopathy [OR = 1.11, 95% CI (1.08, 1.15); P < 0.01], revealing a magnitude smaller than the collective effect of HPT and GD on diabetic retinopathy. The respective appraisals of HPT and GD on diabetic retinopathy were generally comparable. Even after being adjusted for RA, HPT retained a significant impact on diabetic retinopathy (DR/NPDR). Conversely, the MVMR analysis suggested that the association between

			GD and diabetic retinopathy remained unaffected by TOS, but it lost statistical significance after accounting for RA.
15	**Limitations**	Discuss limitations of the study, taking into account the validity of the IV assumptions, other sources of potential bias, and imprecision. Discuss both direction and magnitude of any potential bias and any efforts to address them	It was showed in the Discussion part.
16	**Interpretation**		
	a)	Meaning: Give a cautious overall interpretation of results in the context of their limitations and in comparison with other studies	
	b)	Mechanism: Discuss underlying biological mechanisms that could drive a potential causal relationship between the investigated exposure and the outcome, and whether the gene-environment equivalence assumption is reasonable. Use causal language carefully, clarifying that IV estimates may provide causal effects only under certain assumptions	
	c)	Clinical relevance: Discuss whether the results have clinical or public policy relevance, and to what extent they inform effect sizes of possible interventions	MR results are also characterized by uncertainty, but conclusions can still be drawn.
17	**Generalizability**	Discuss the generalizability of the study results (a) to other populations, (b) across other exposure periods/timings, and (c) across other levels of exposure	The restriction of this study to populations of European ancestry, while reducing potential bias due to demographics, limits the generalizability of the MR results to other populations.
	**OTHER INFORMATION**		
18	**Funding**	Describe sources of funding and the role of funders in the present study and, if applicable, sources of funding for the databases and original study or studies on which the present study is based	This article has not received any funding.
19	**Data and data sharing**	Provide the data used to perform all analyses or report where and how the data can be accessed, and reference these sources in the article. Provide the statistical code needed to reproduce the results in the article, or report whether the code is publicly accessible and if so, where	In the method section, we provide access to all the data sources. Our teams never agree to provided the codes of R, but if readers want to original codes, readers can contact Corresponding author.
20	**Conflicts of Interest**	All authors should declare all potential conflicts of interest	This article has not received any funding.

This checklist is copyrighted by the Equator Network under the Creative Commons Attribution 3.0 Unported (CC BY 3.0) license.

1. Skrivankova VW, Richmond RC, Woolf BAR, Yarmolinsky J, Davies NM, Swanson SA, et al. Strengthening the Reporting of Observational Studies in Epidemiology using Mendelian Randomization (STROBE-MR) Statement. JAMA. 2021;under review.

2. Skrivankova VW, Richmond RC, Woolf BAR, Davies NM, Swanson SA, VanderWeele TJ, et al. Strengthening the Reporting of Observational Studies in Epidemiology using Mendelian Randomisation (STROBE-MR): Explanation and Elaboration. BMJ. 2021;375:n2233.
