# Supplementary material for: Genetically mimicked effects of thyroid dysfunction on diabetic retinopathy risk: a 2-sample univariable and multivariable Mendelian randomization study
Source: Front Endocrinol (Lausanne). 2024 Oct 21;15:1374254. doi: 10.3389/fendo.2024.1374254 (PMC11532173; doi:10.3389/fendo.2024.1374254)

Supplemental Figure 1A. Result for the RadialMR analysis in the SNPs (RadialMR plot): TOS -DBR.

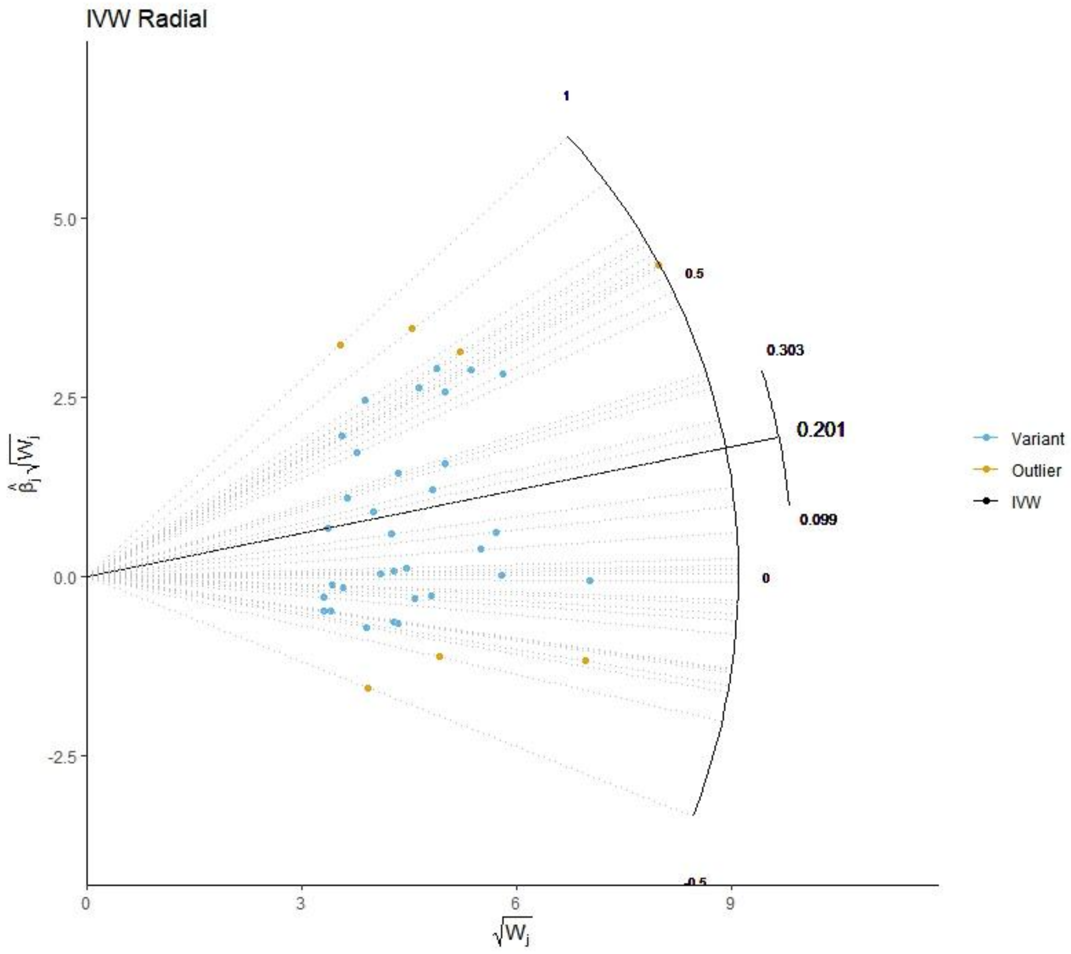

Supplemental Figure 1B. Result for the RadialMR analysis in the SNPs (RadialMR plot): TOS- DR.

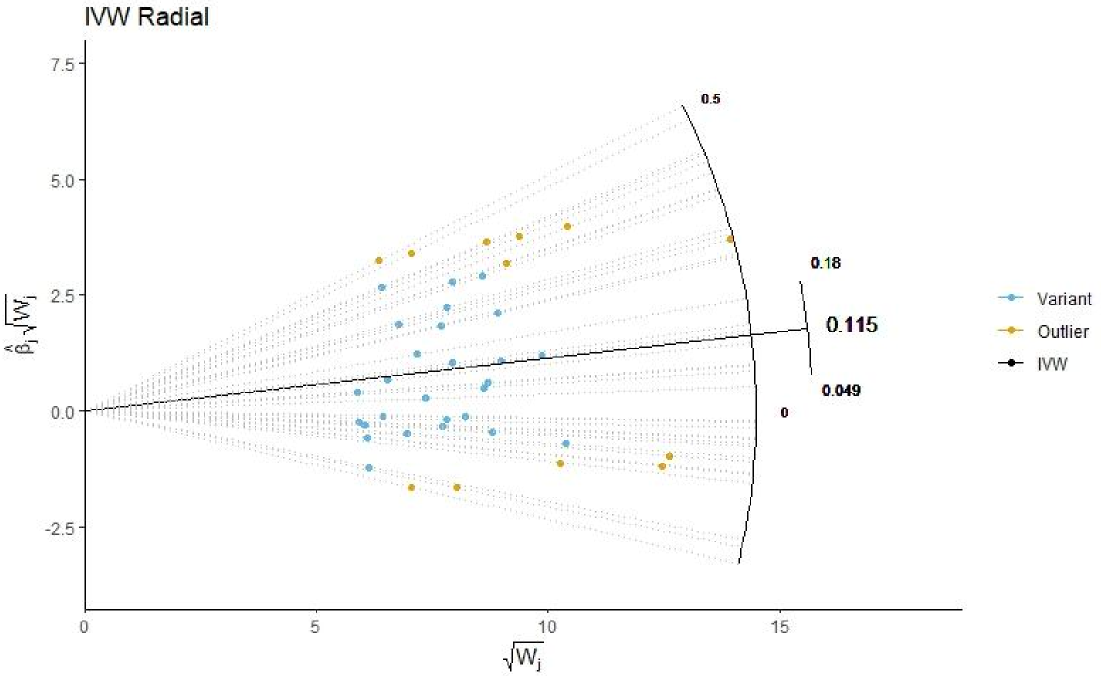

Supplemental Figure 1C. Result for the RadialMR analysis in the SNPs (RadialMR plot): TOS-NPDR.

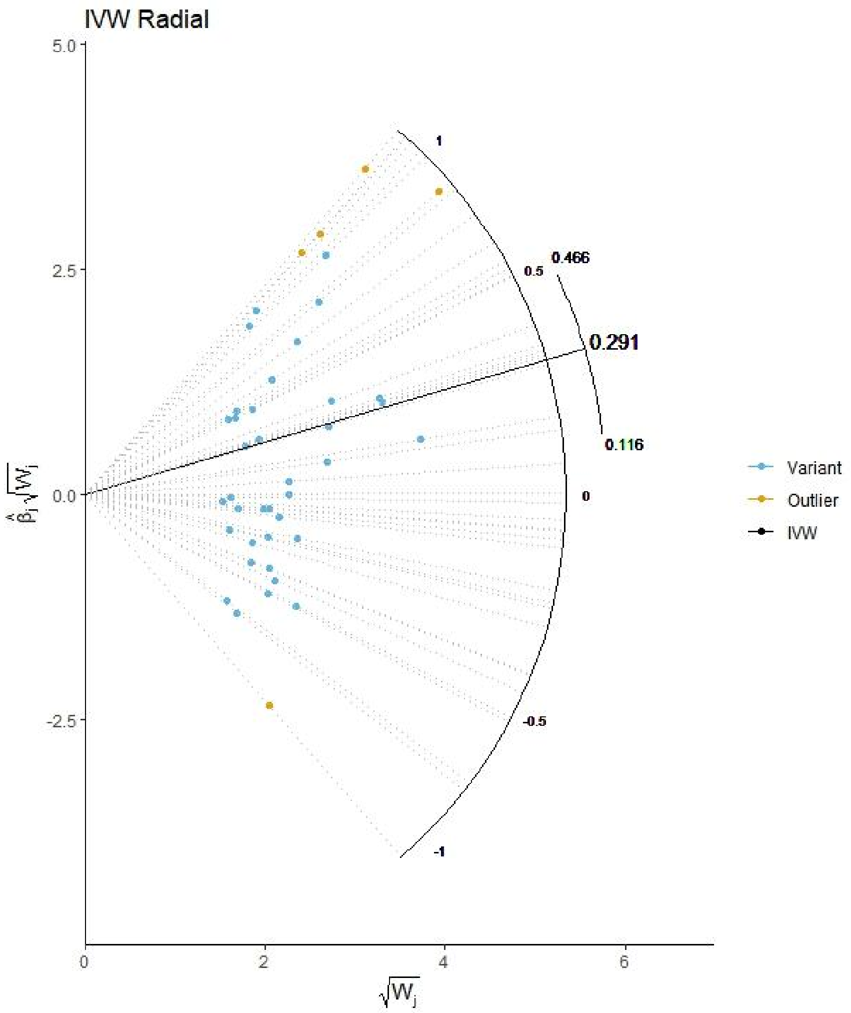

Supplemental Figure 1D. Result for the RadialMR analysis in the SNPs (RadialMR plot): TOS-PDR.

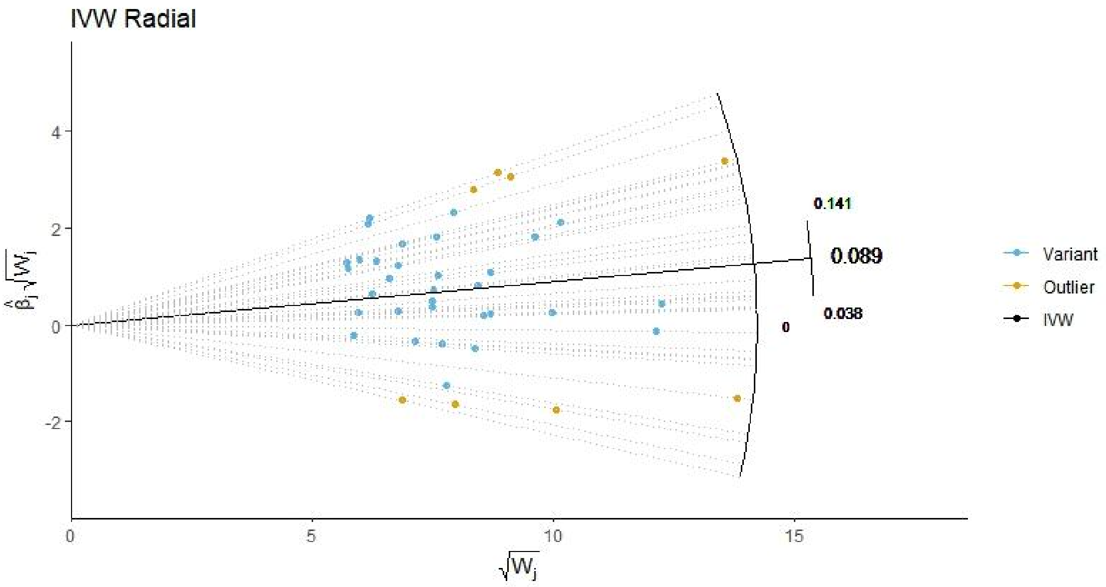

Supplemental Figure 2A. Result for the RadialMR analysis in the SNPs (RadialMR plot): HPT-DBR.

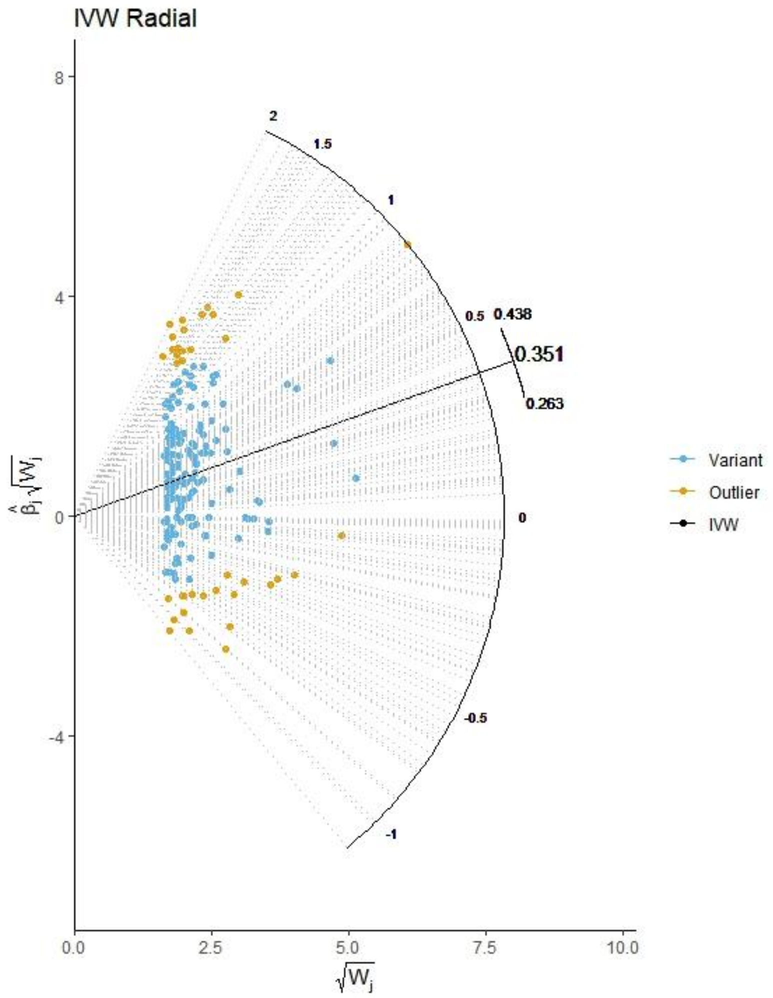

Supplemental Figure 2B. Result for the RadialMR analysis in the SNPs (RadialMR plot): HPT-DR.

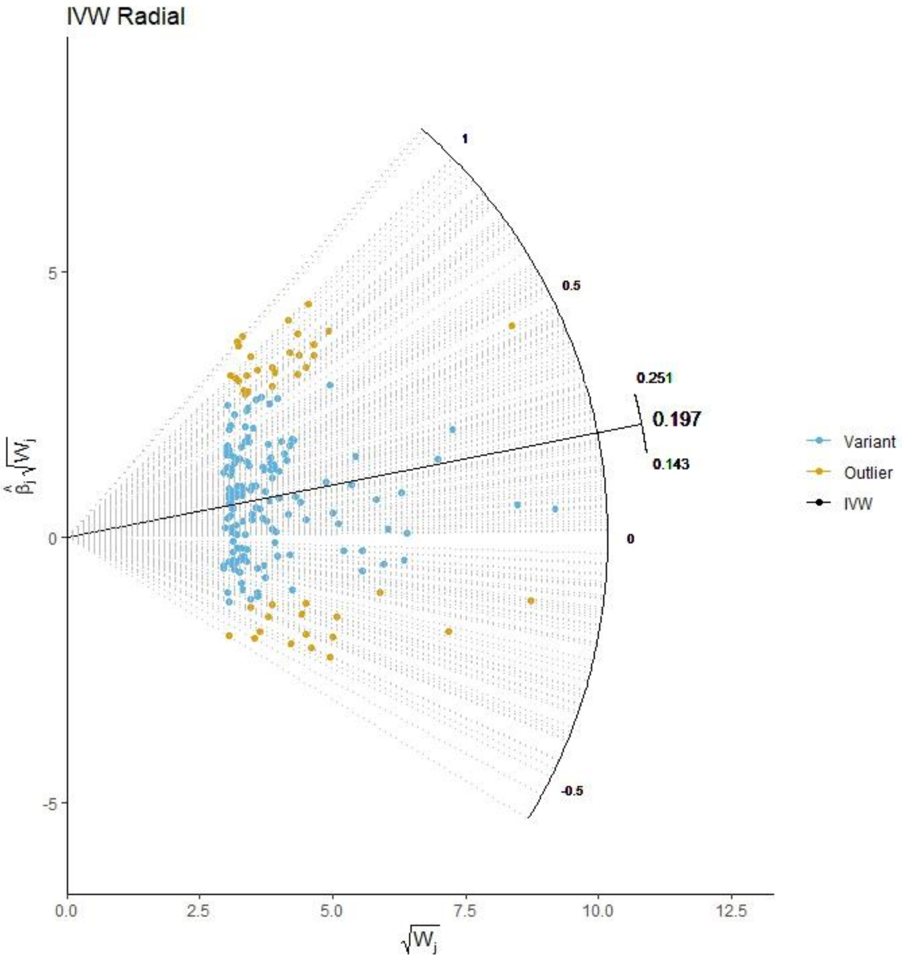

Supplemental Figure 2C. Result for the RadialMR analysis in the SNPs (RadialMR plot):HPT-NPDR.

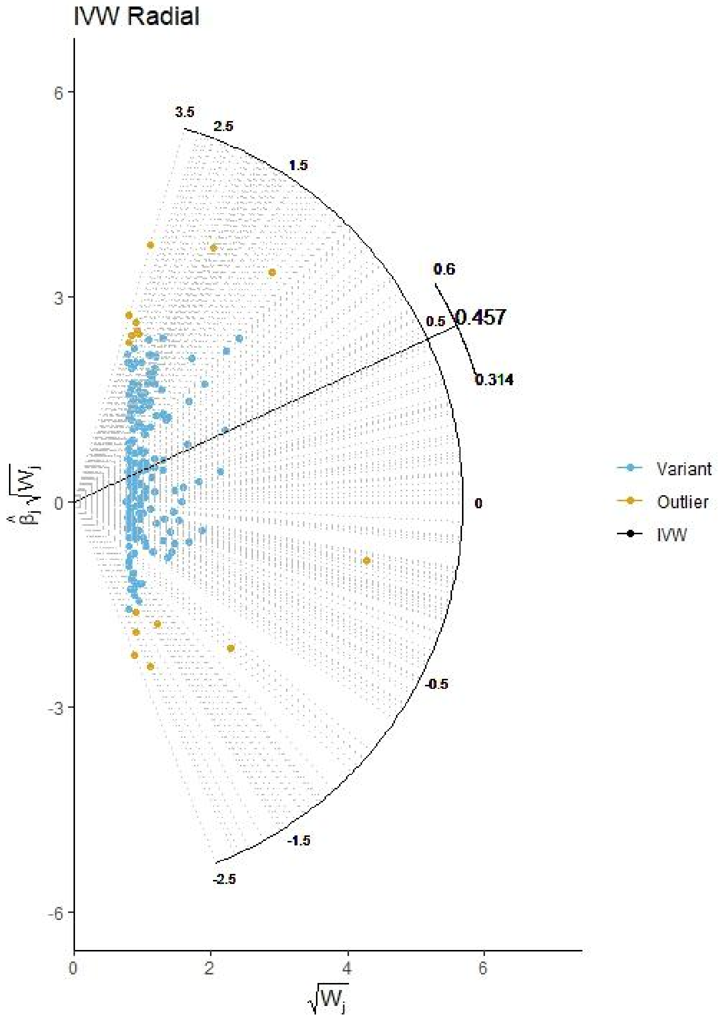

Supplemental Figure 2D. Result for the RadialMR analysis in the SNPs (RadialMR plot):HPT-PDR.

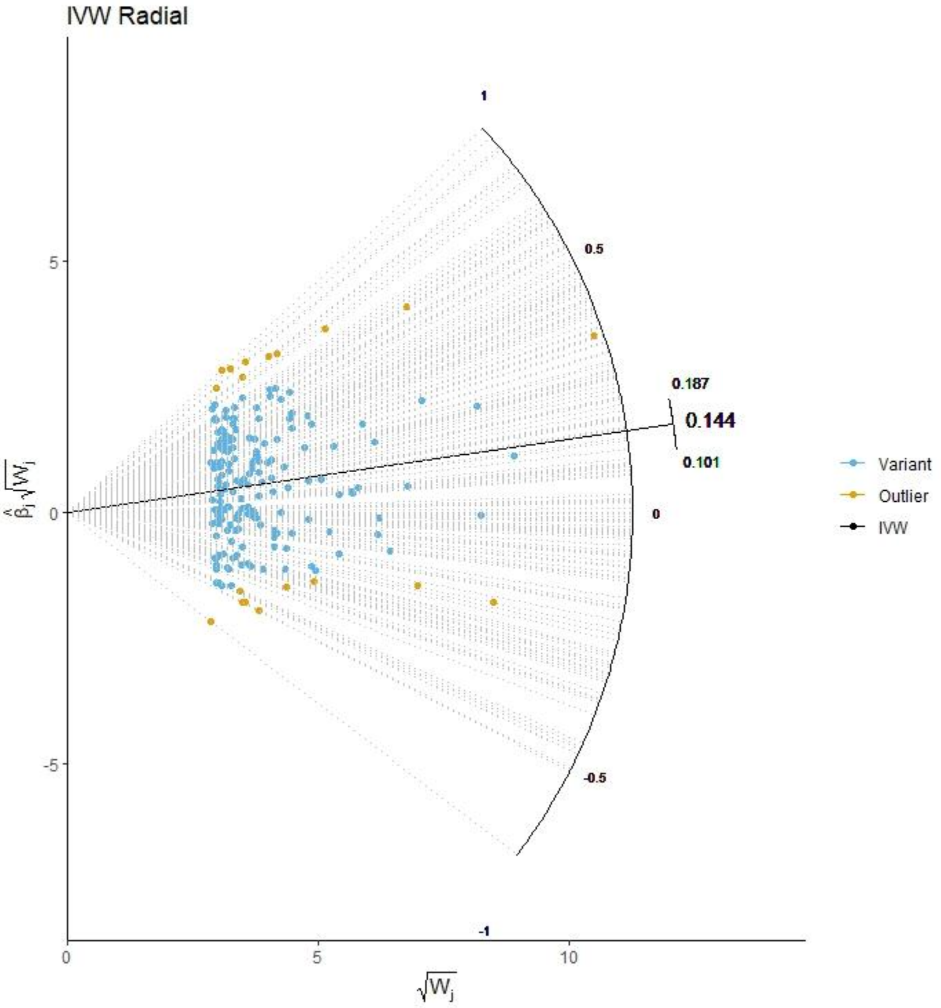

Supplemental Figure 3A. Result for the RadialMR analysis in the SNPs (RadialMR plot): GD-DBR.

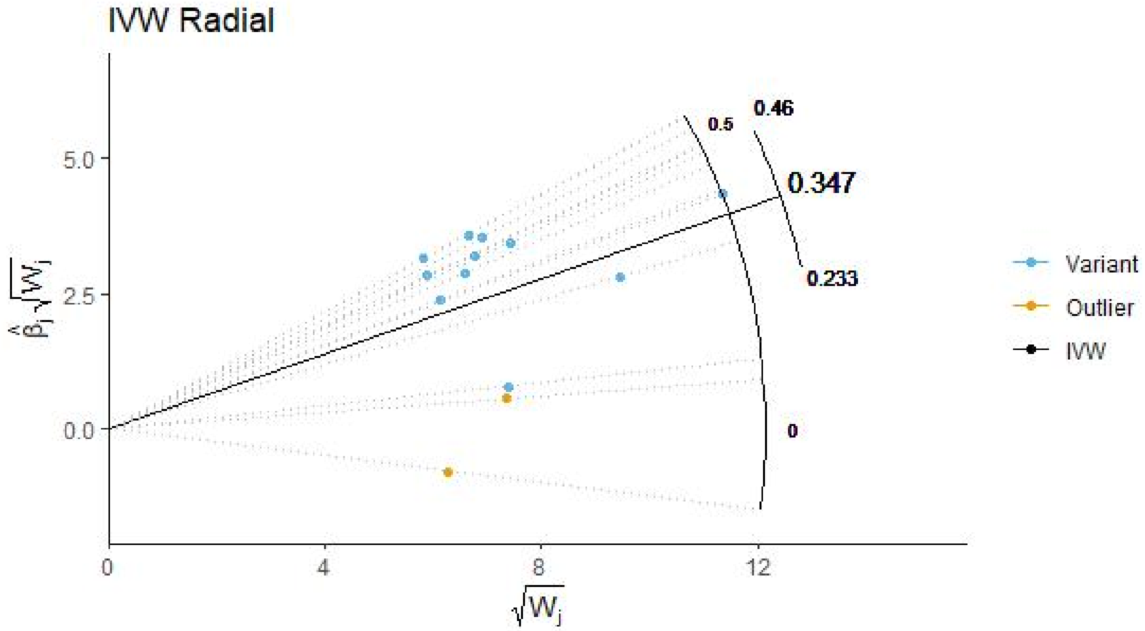

Supplemental Figure 3B. Result for the RadialMR analysis in the SNPs (RadialMR plot): GD-DR.

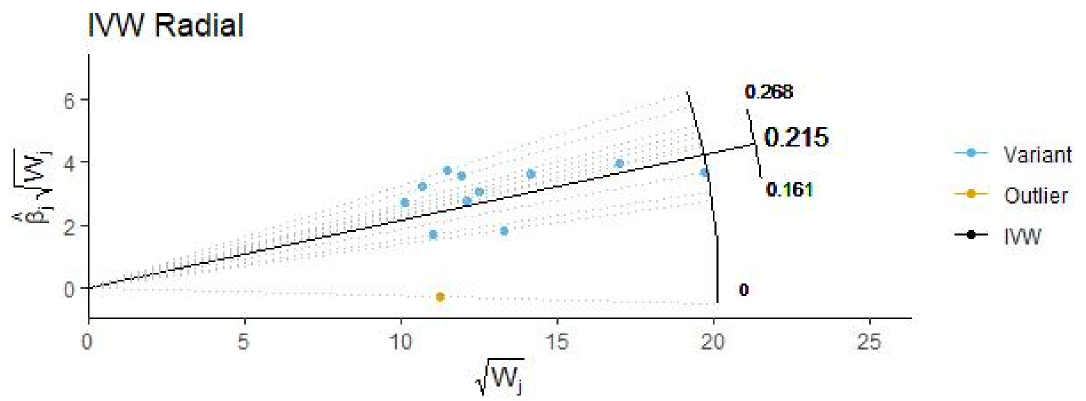

Supplemental Figure 3C. Result for the RadialMR analysis in the SNPs (RadialMR plot): GD-NPDR.

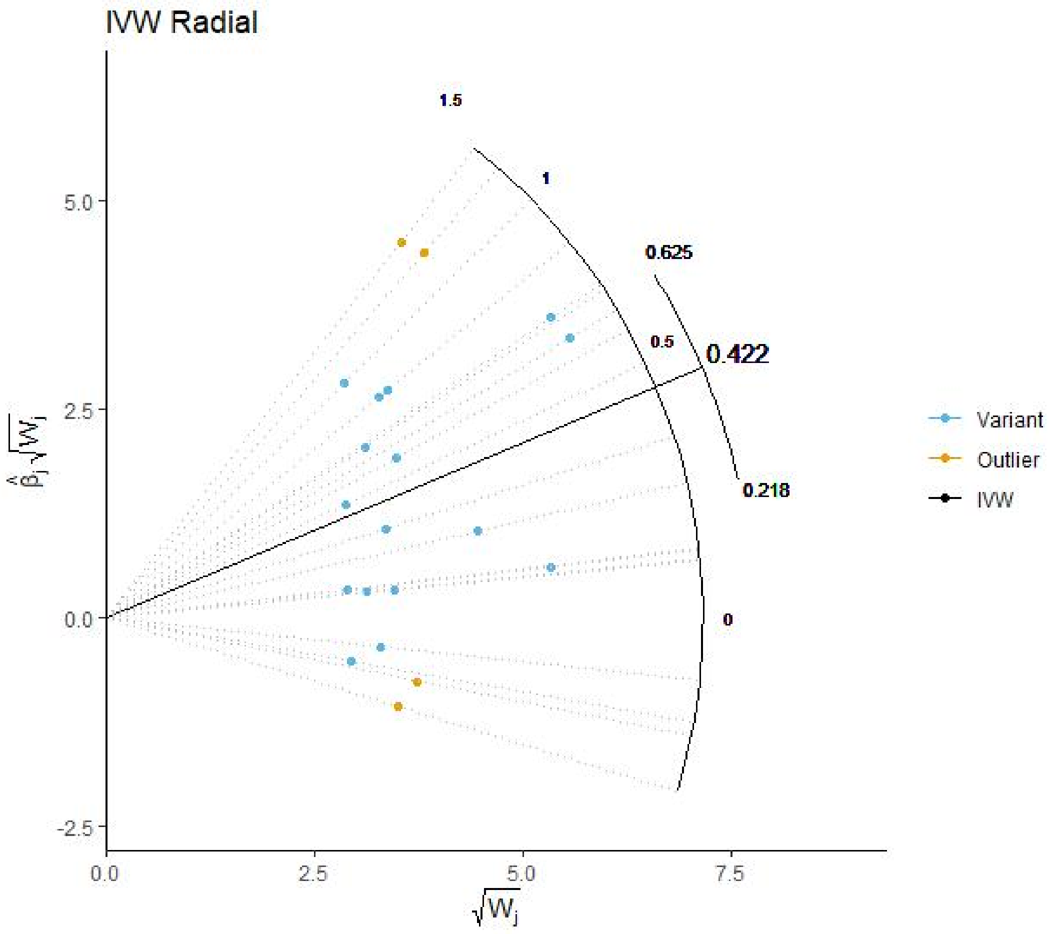

Supplemental Figure 3D. Result for the RadialMR analysis in the SNPs (RadialMR plot): GD-PDR.

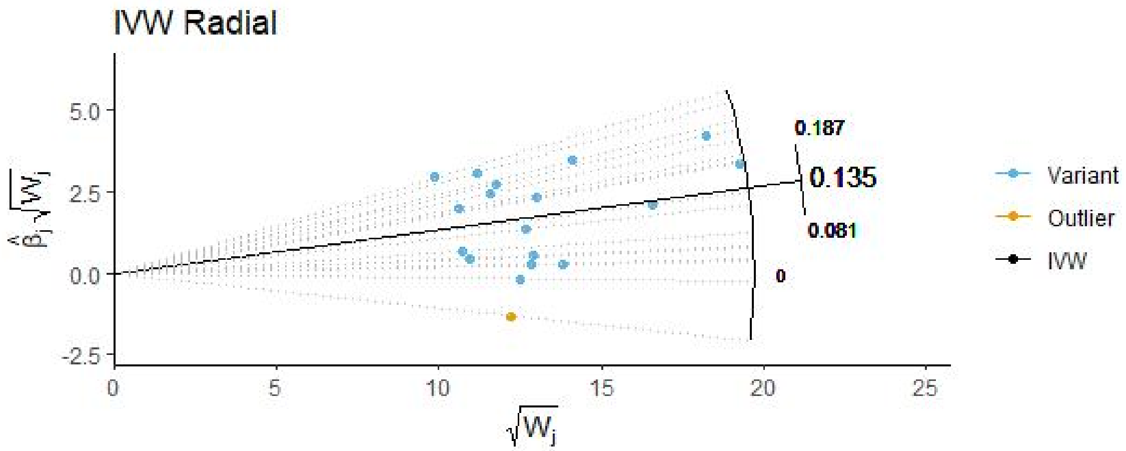

Supplemental Figure 4A. leave-one-out of the MR analysis: TOS -DBR.

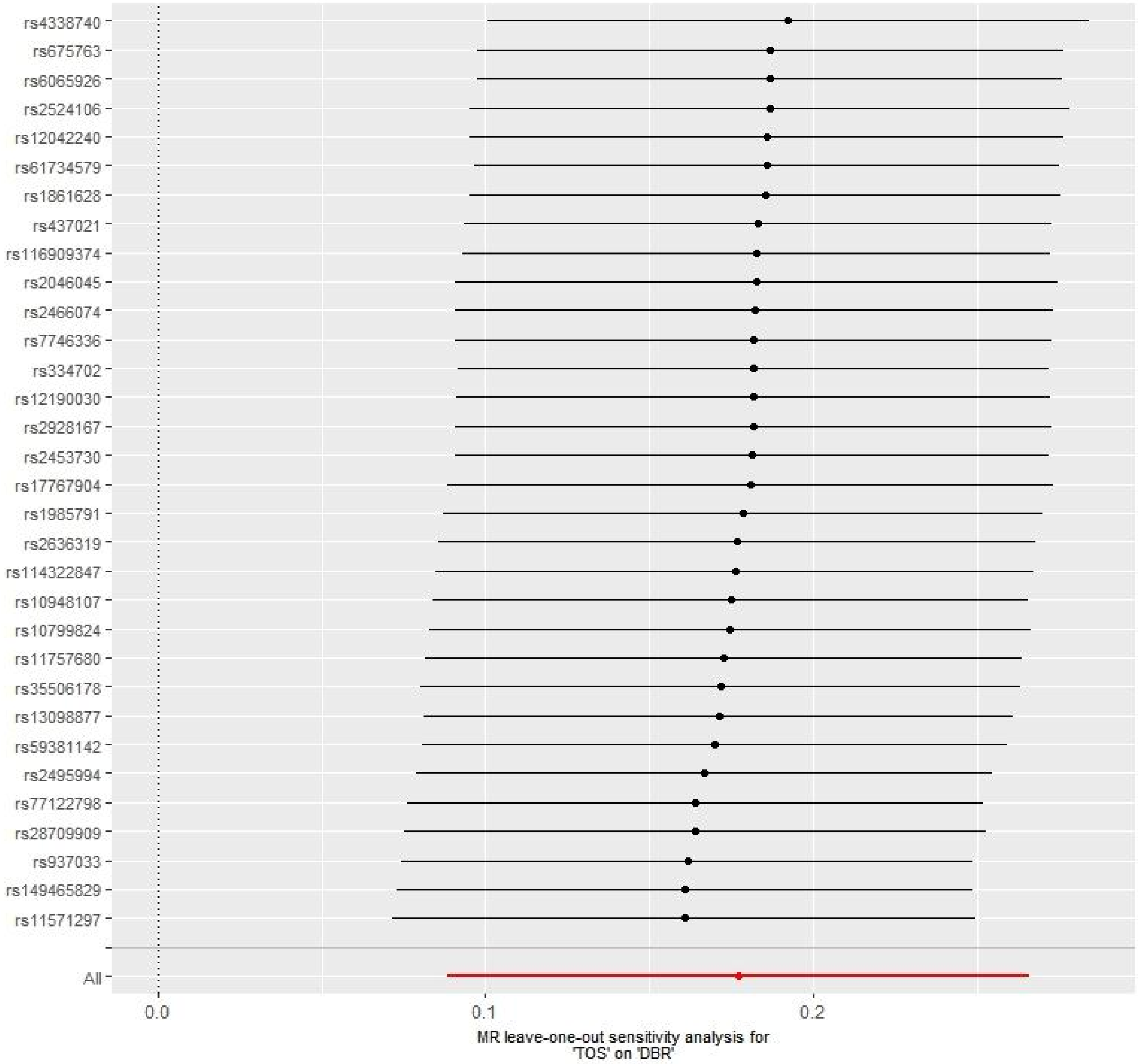

Supplemental Figure 4B. leave-one-out of the MR analysis: TOS- DR.

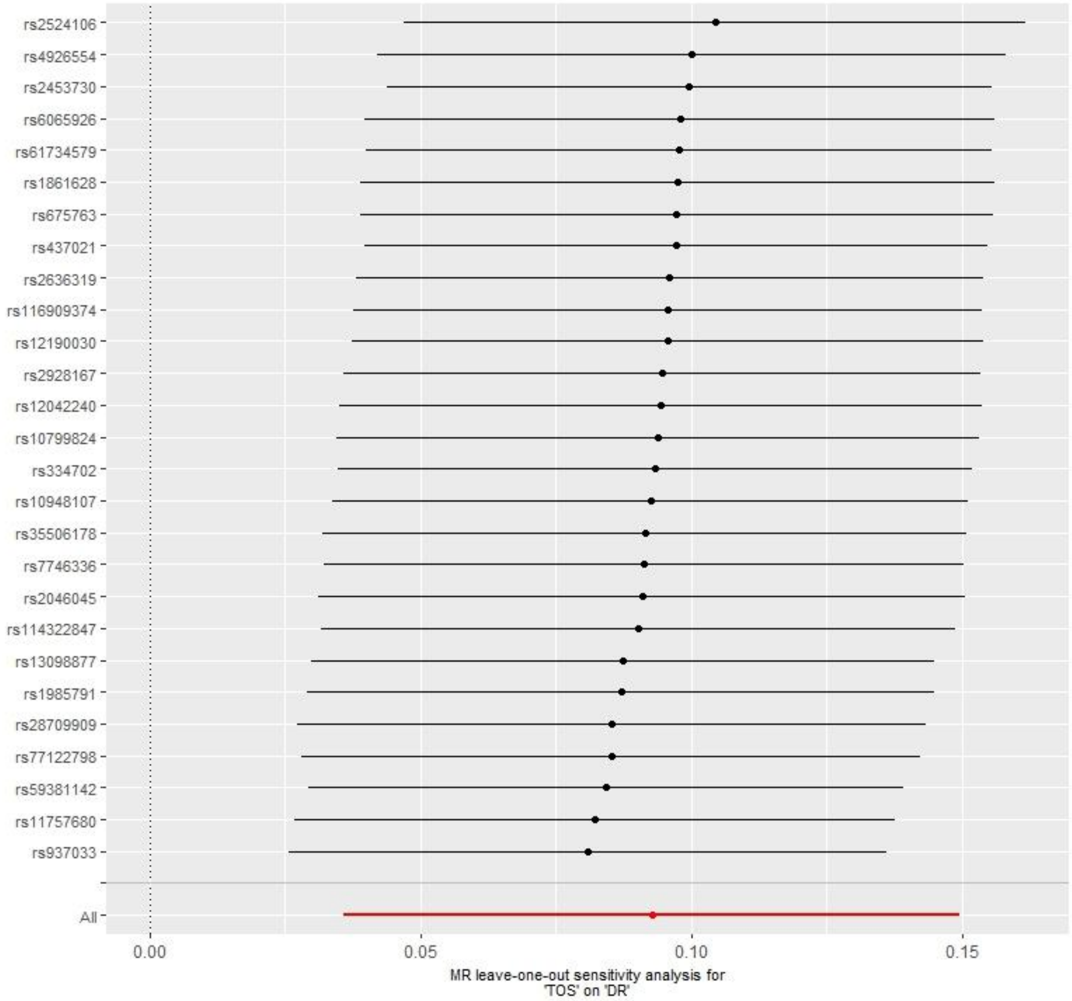

Supplemental Figure 4C. leave-one-out of the MR analysis: TOS-NPDR.

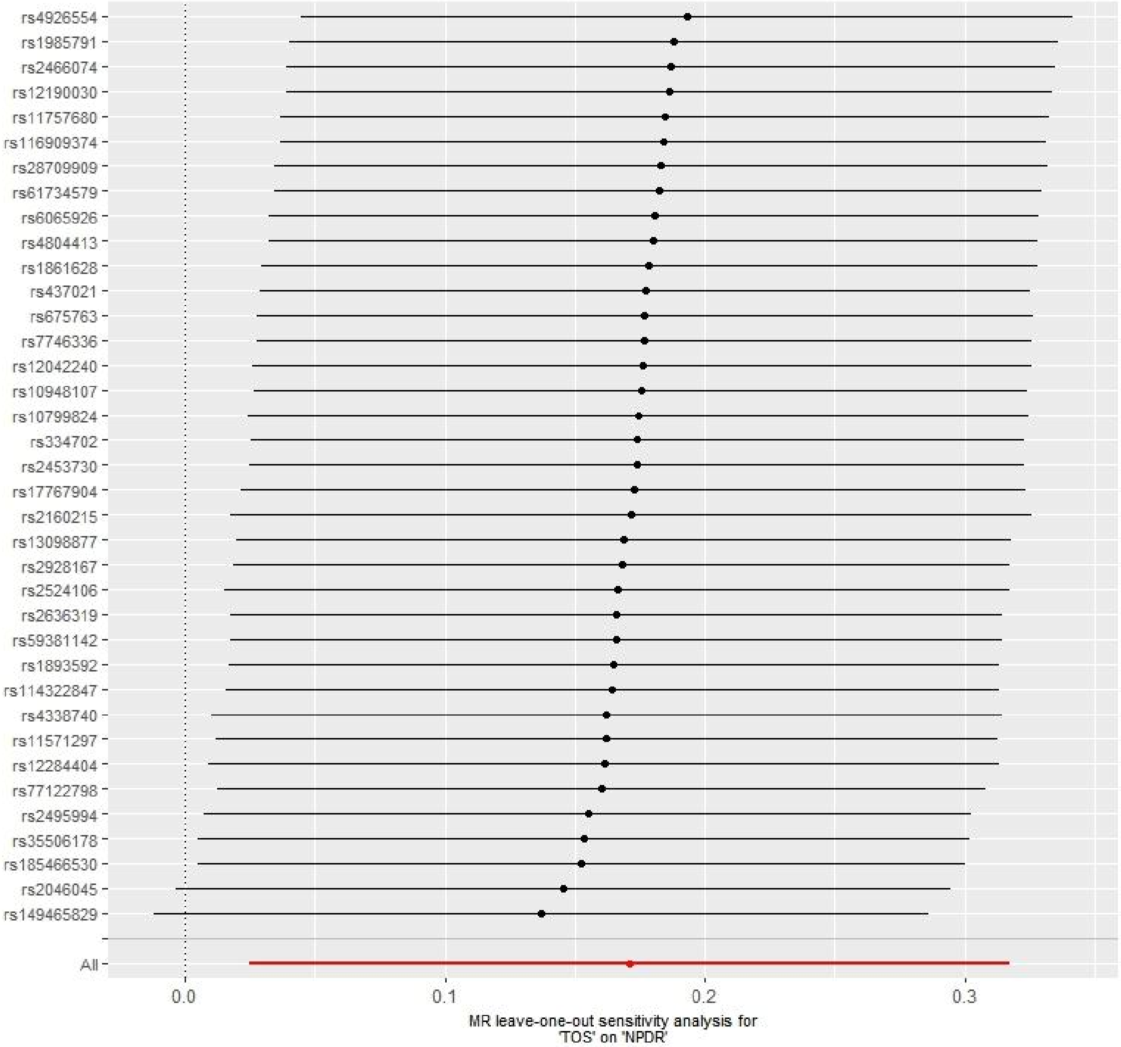

Supplemental Figure 4D. leave-one-out of the MR analysis: TOS-PDR.

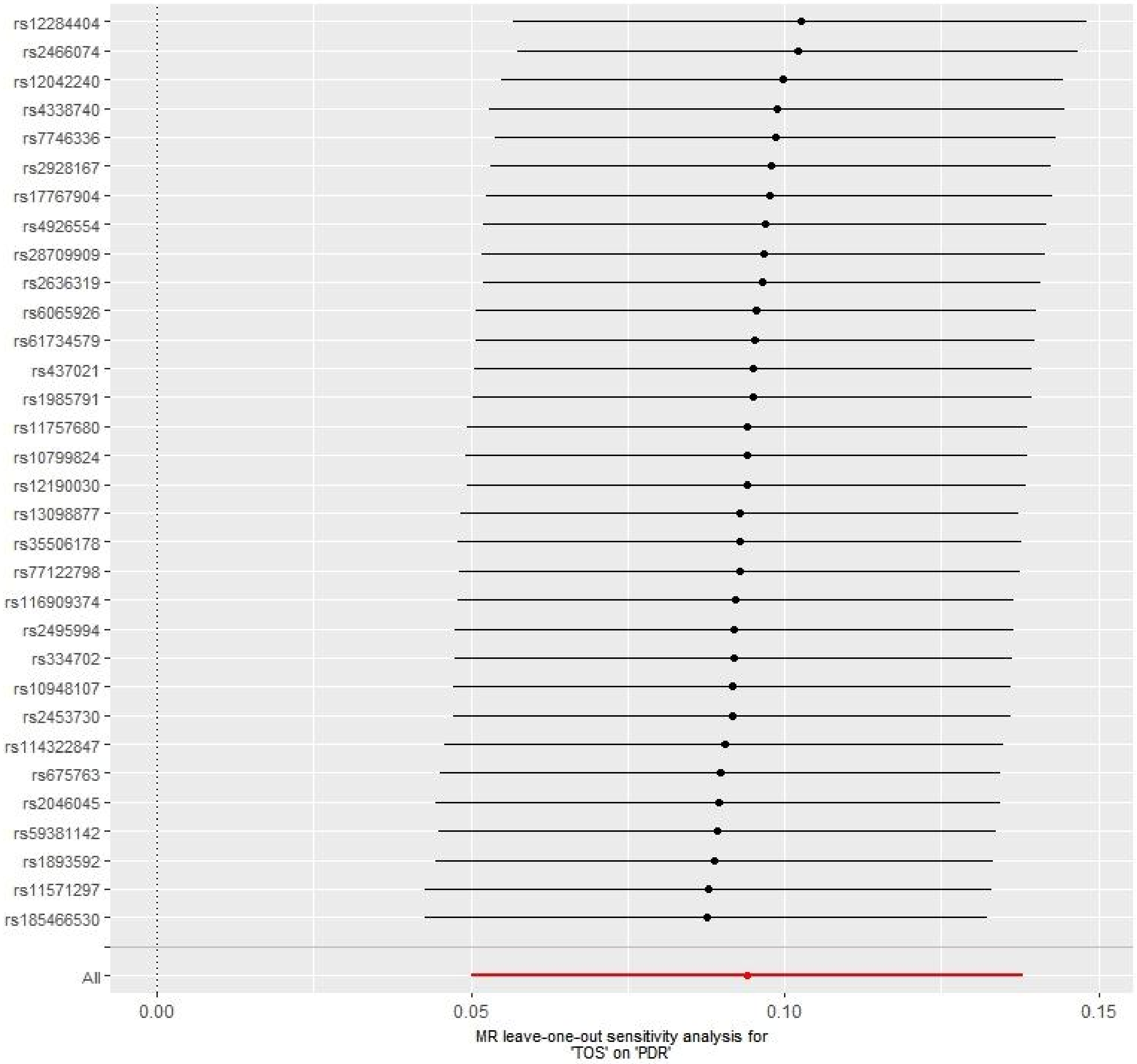

Supplemental Figure 5A. leave-one-out of the MR analysis: HPT-DBR.

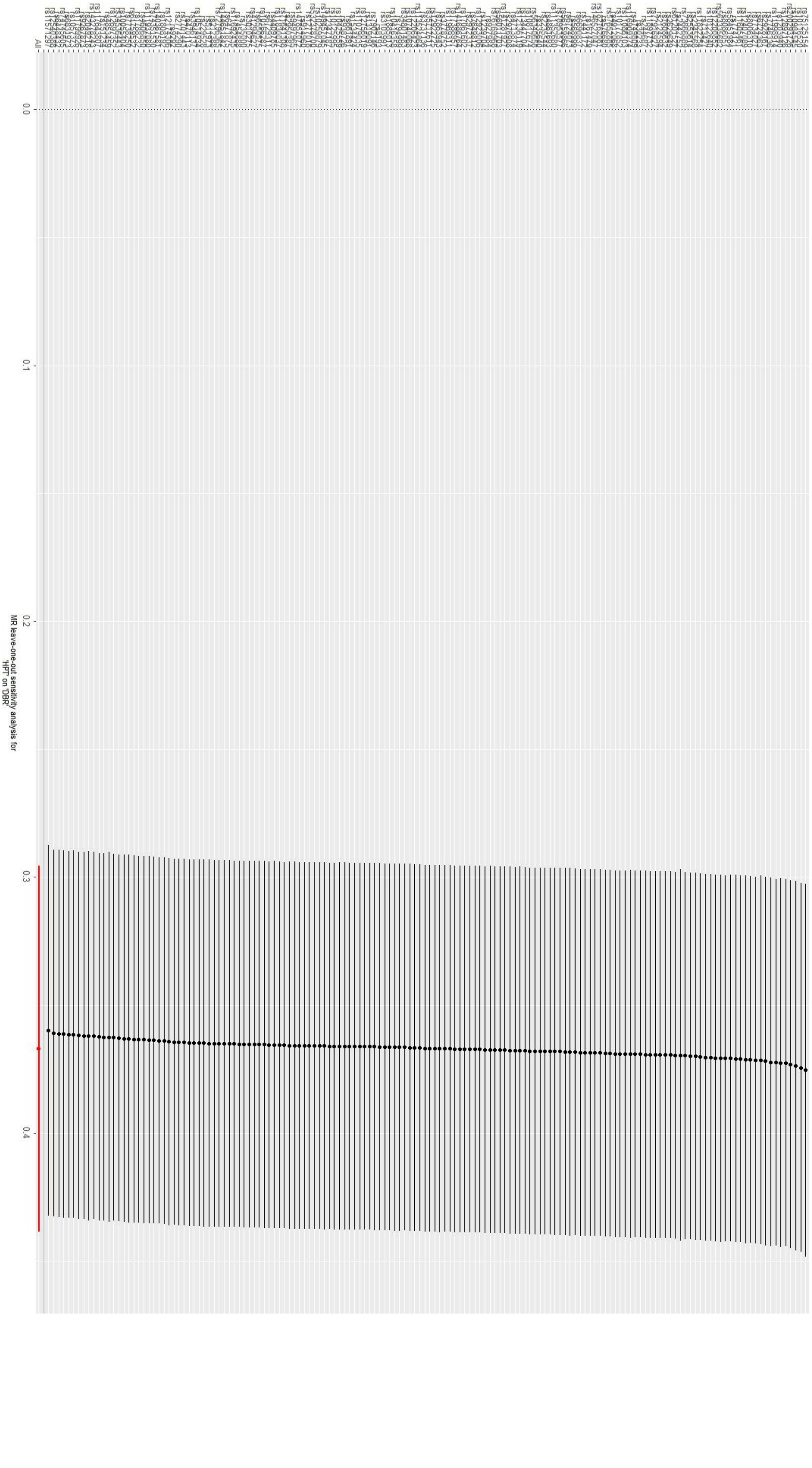

Supplemental Figure 5B. leave-one-out of the MR analysis: HPT-DR.

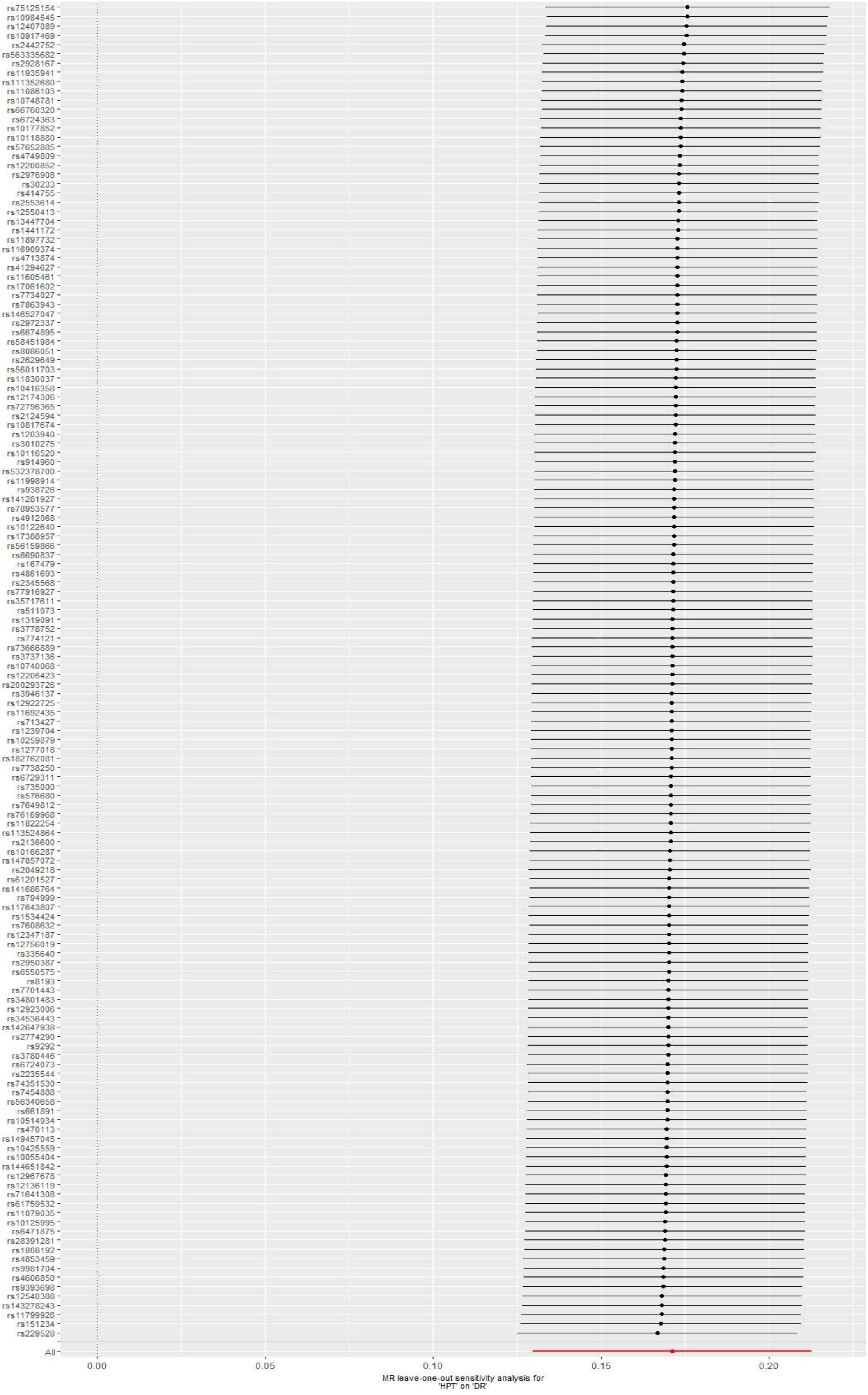

Supplemental Figure 5C. leave-one-out of the MR analysis: HPT-NPDR.

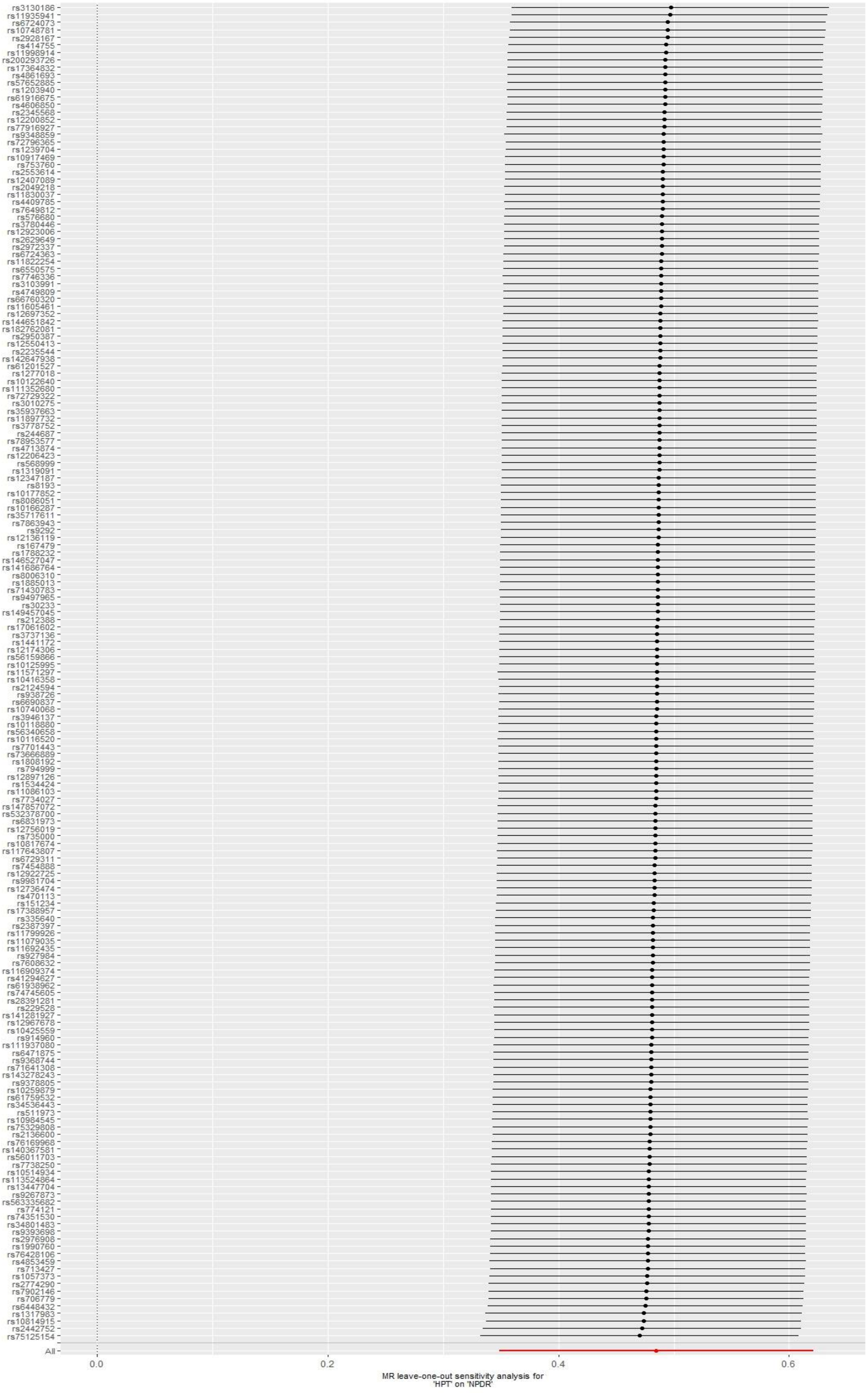

Supplemental Figure 5D. leave-one-out of the MR analysis: HPT-PDR.

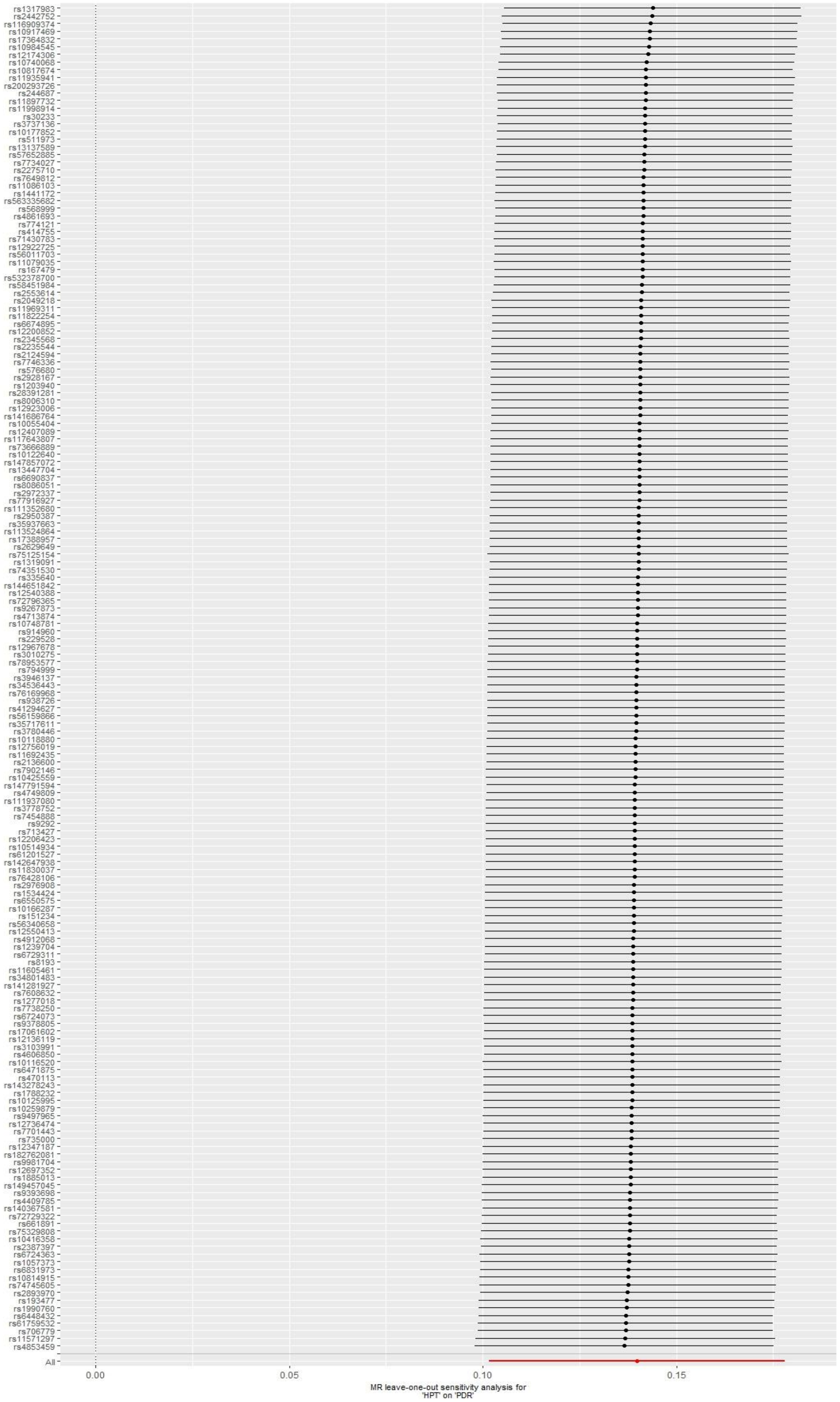

Supplemental Figure 6A. leave-one-out of the MR analysis: GD-DBR.

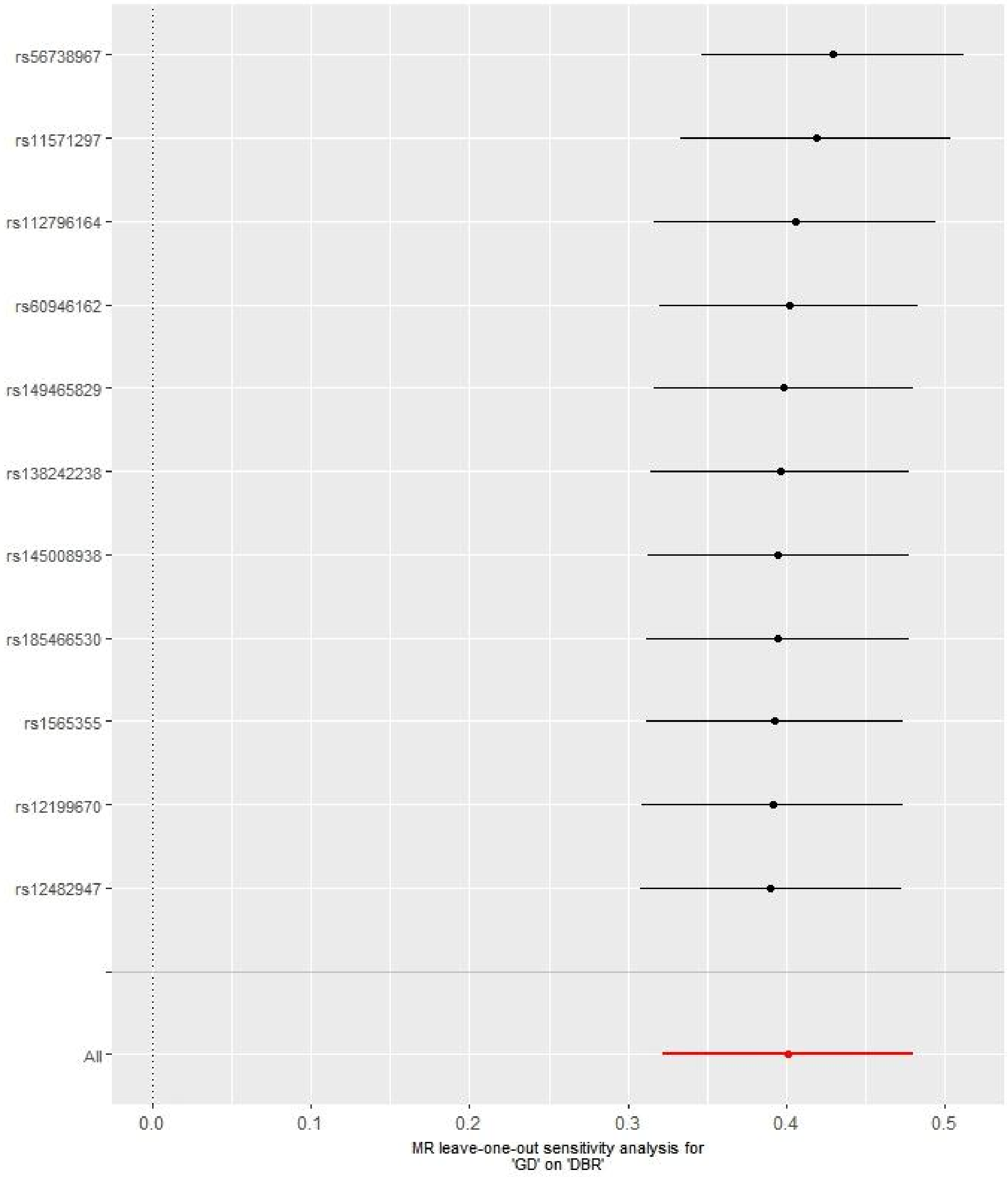

Supplemental Figure 6B. leave-one-out of the MR analysis: GD-DR.

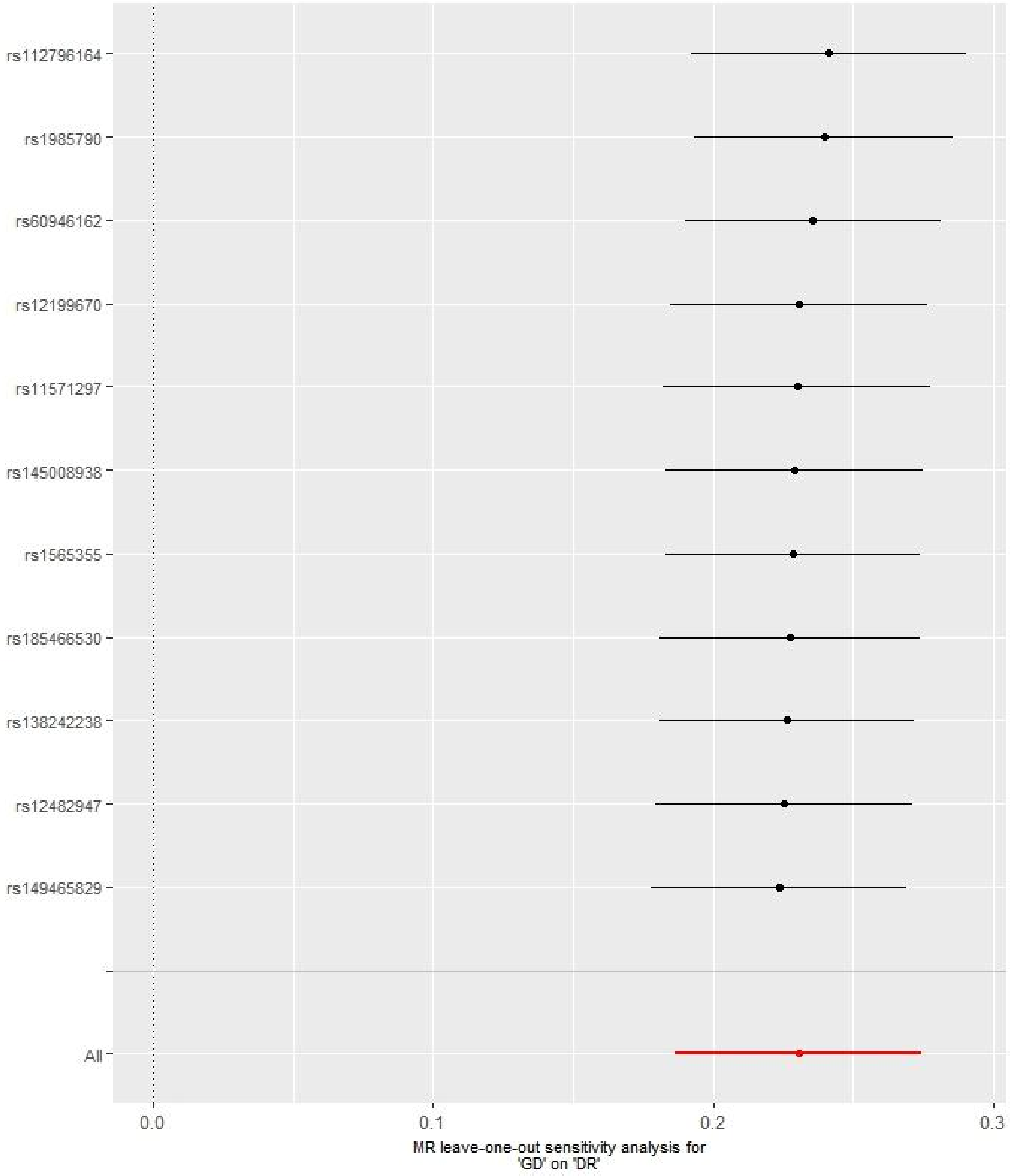

Supplemental Figure 6C. leave-one-out of the MR analysis: GD-NPDR.

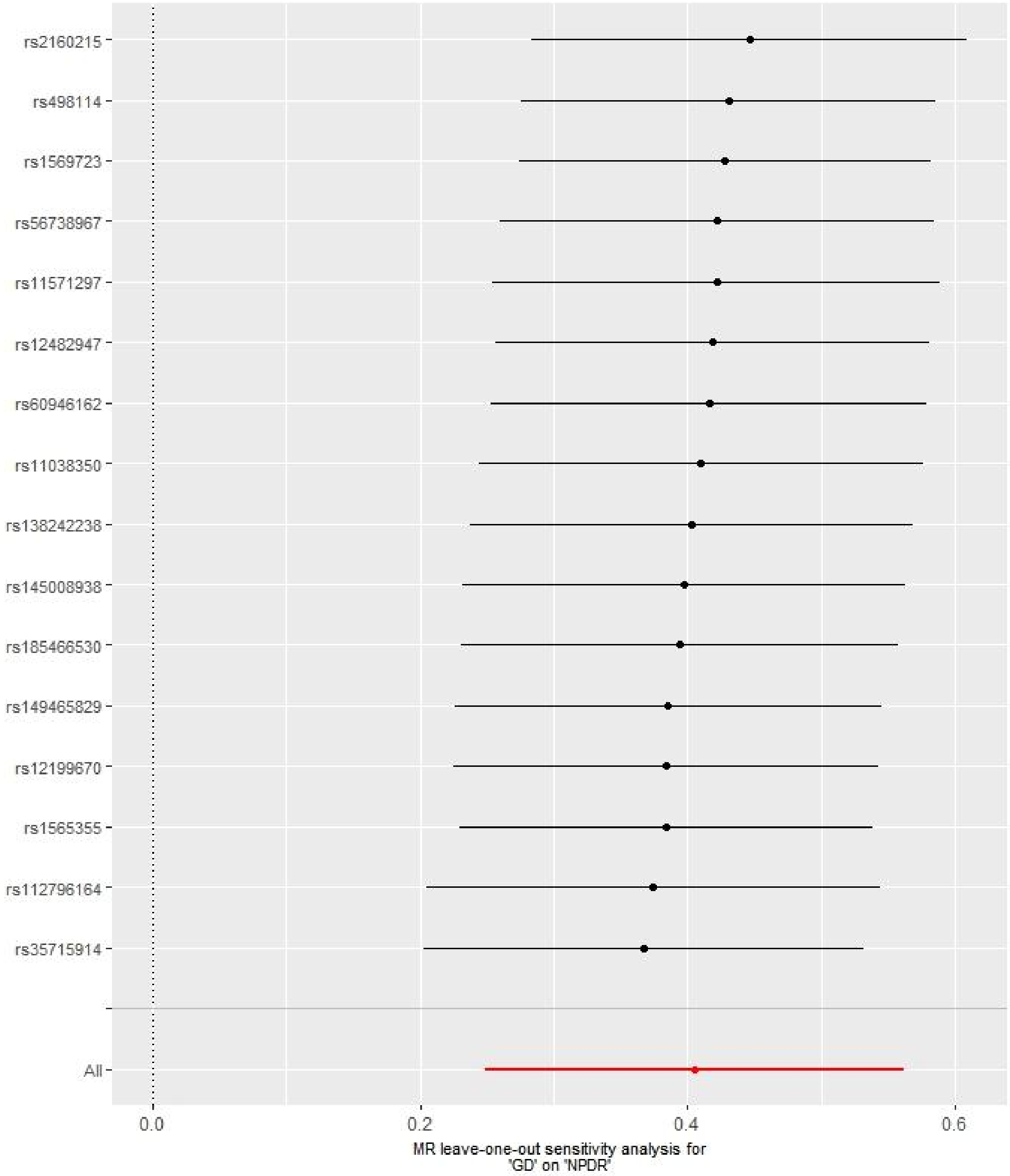

Supplemental Figure 6D. leave-one-out of the MR analysis: GD-PDR.

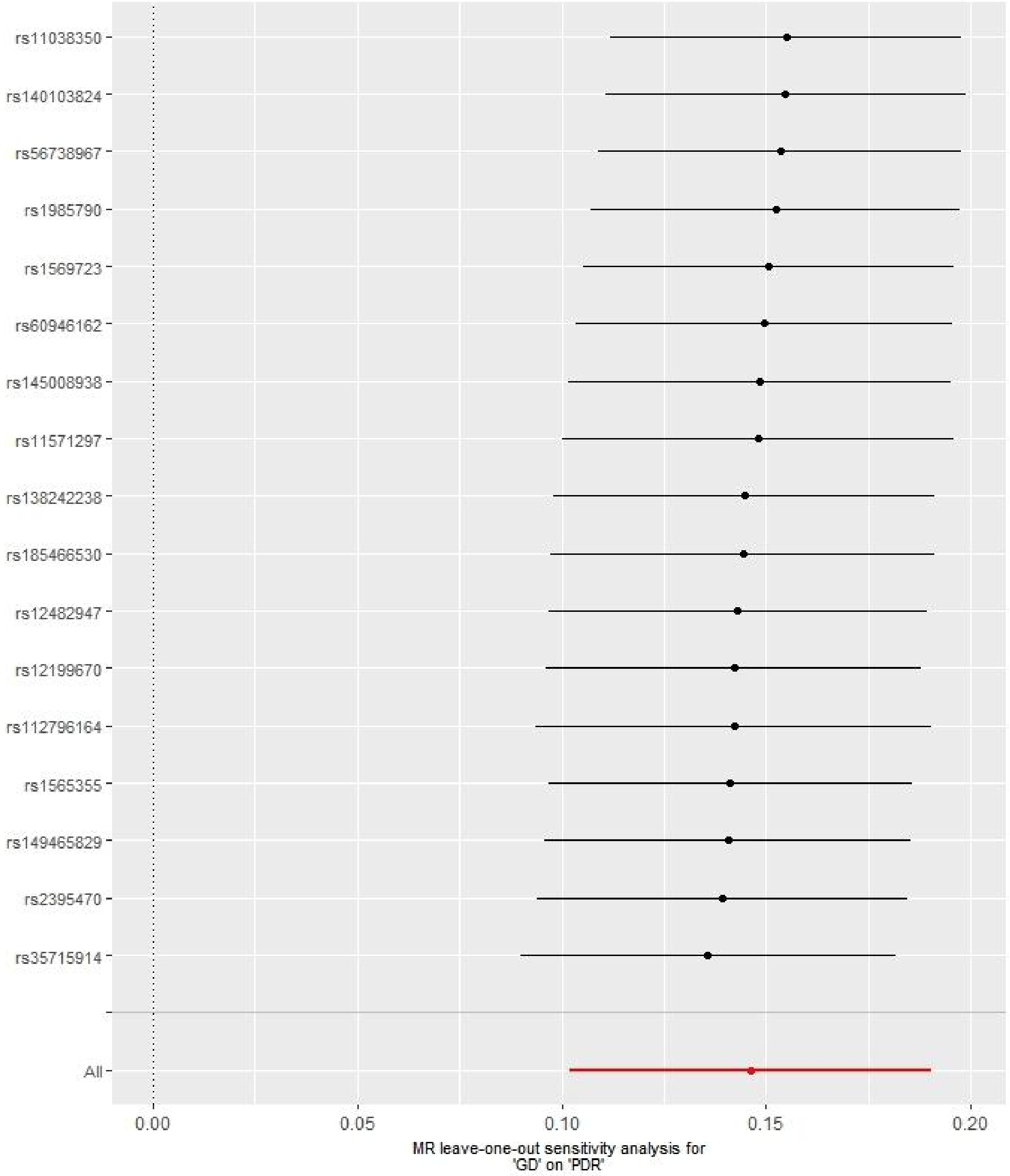

Supplemental Figure 7A. Scatter plots of the MR analysis: TOS -DBR.

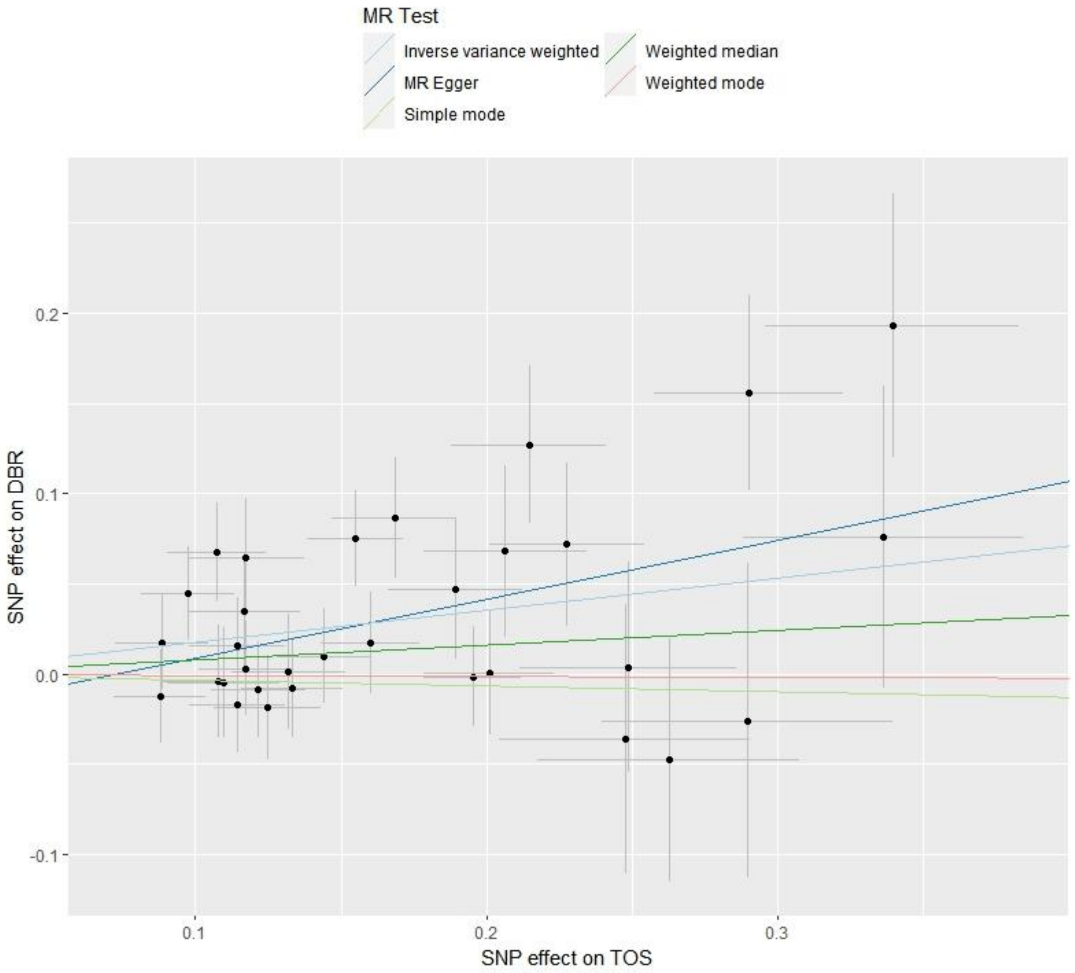

Supplemental Figure 7B. Scatter plots of the MR analysis: TOS- DR.

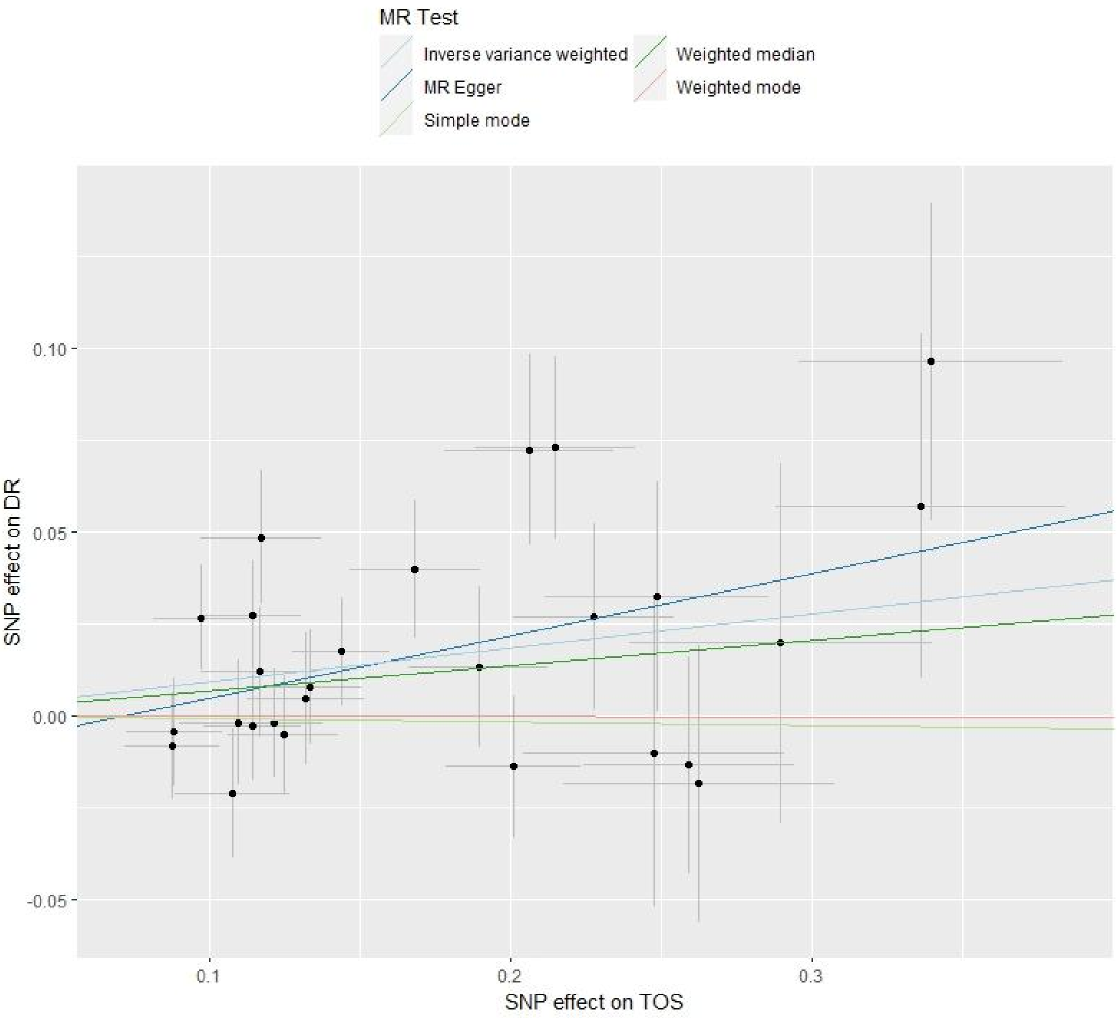

Supplemental Figure 7C. Scatter plots of the MR analysis: TOS-NPDR.

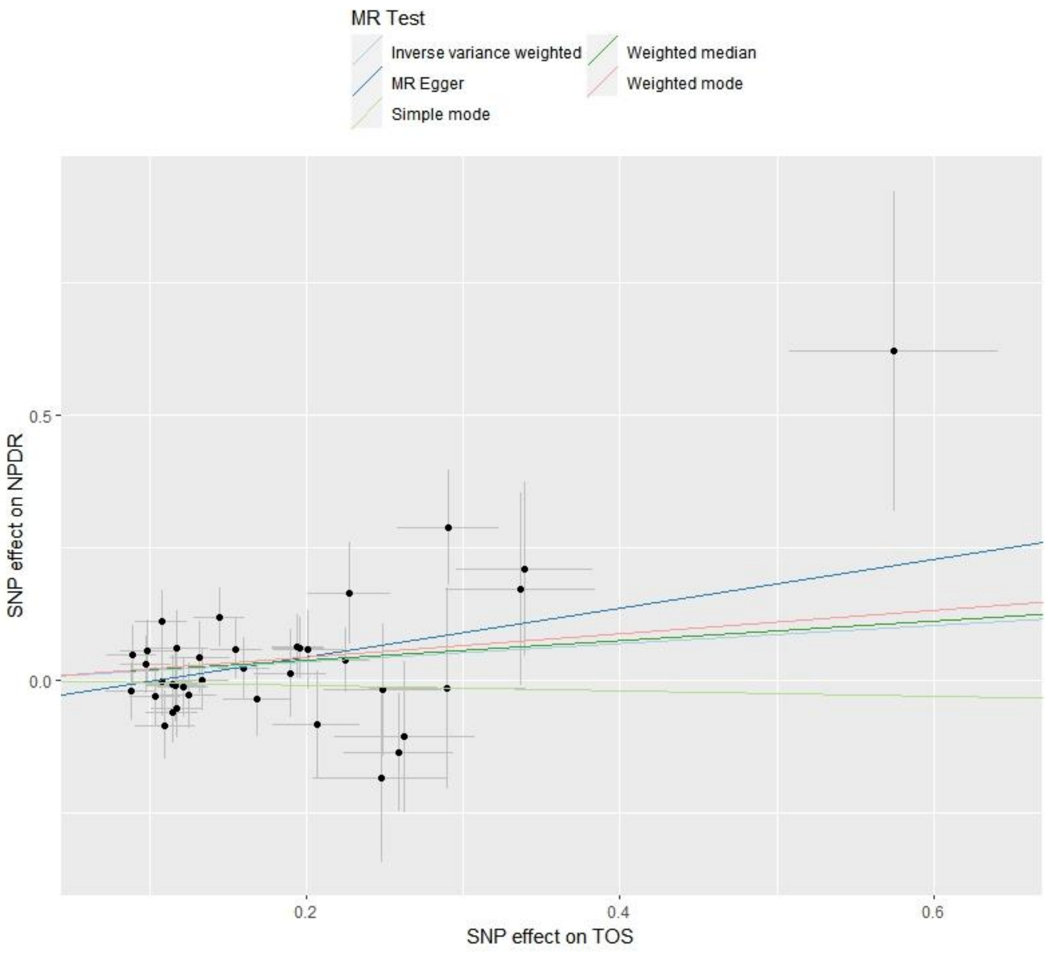

Supplemental Figure 7D. Scatter plots of the MR analysis: TOS-PDR.

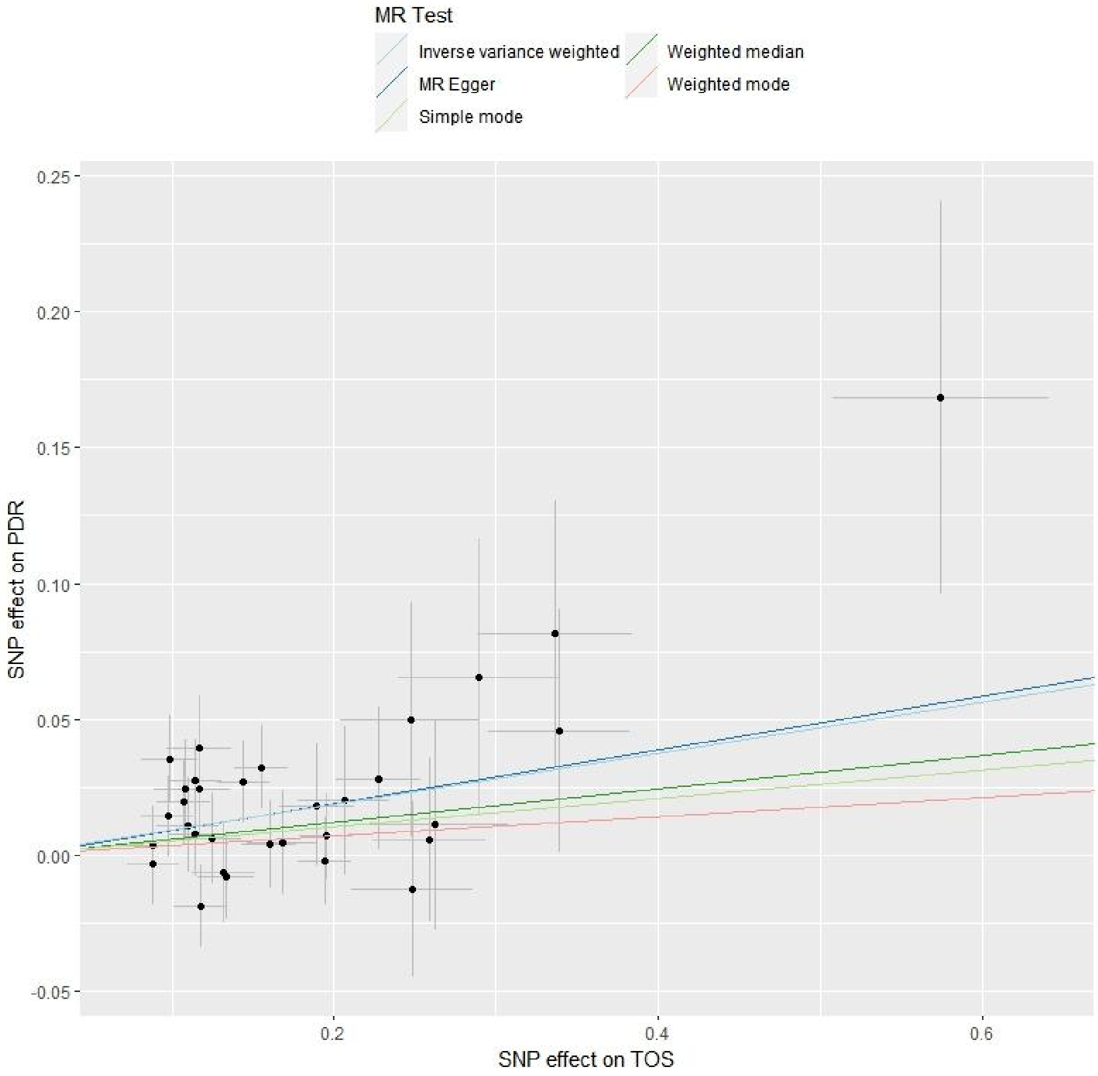

Supplemental Figure 8A. Scatter plots of the MR analysis: HPT-DBR.

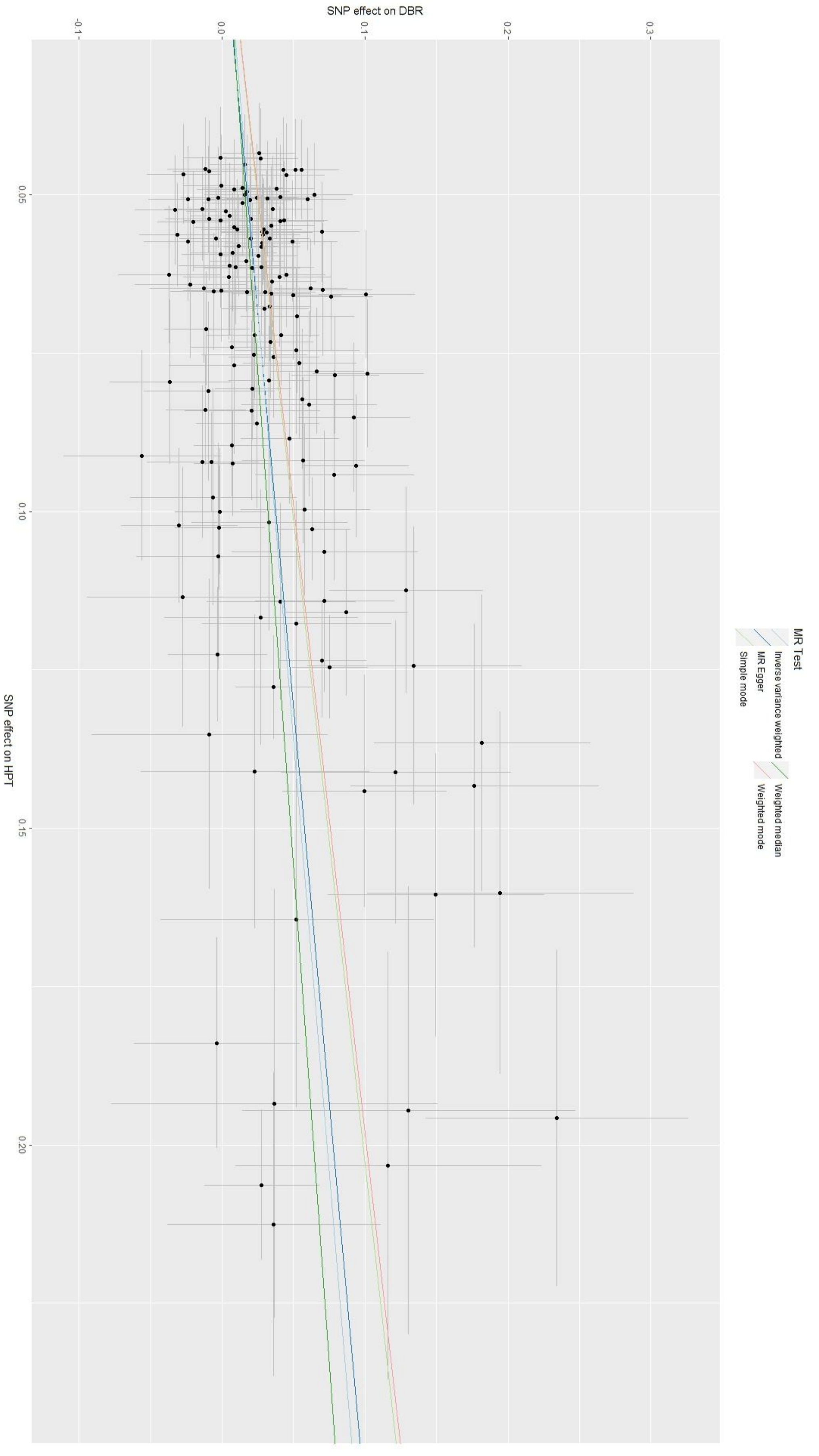

Supplemental Figure 8B. Scatter plots of the MR analysis: HPT-DR.

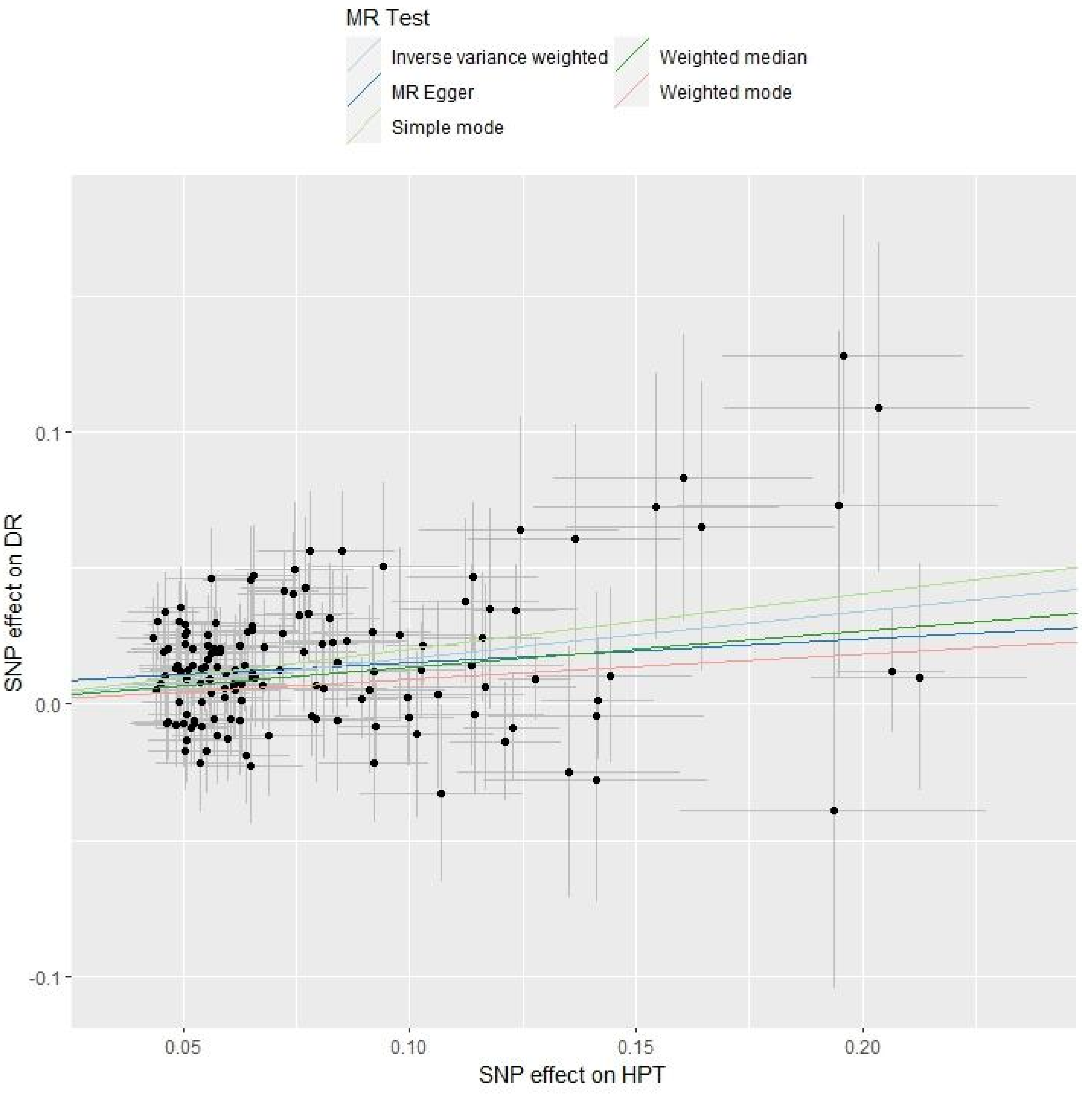

Supplemental Figure 8C. Scatter plots of the MR analysis: HPT-NPDR.

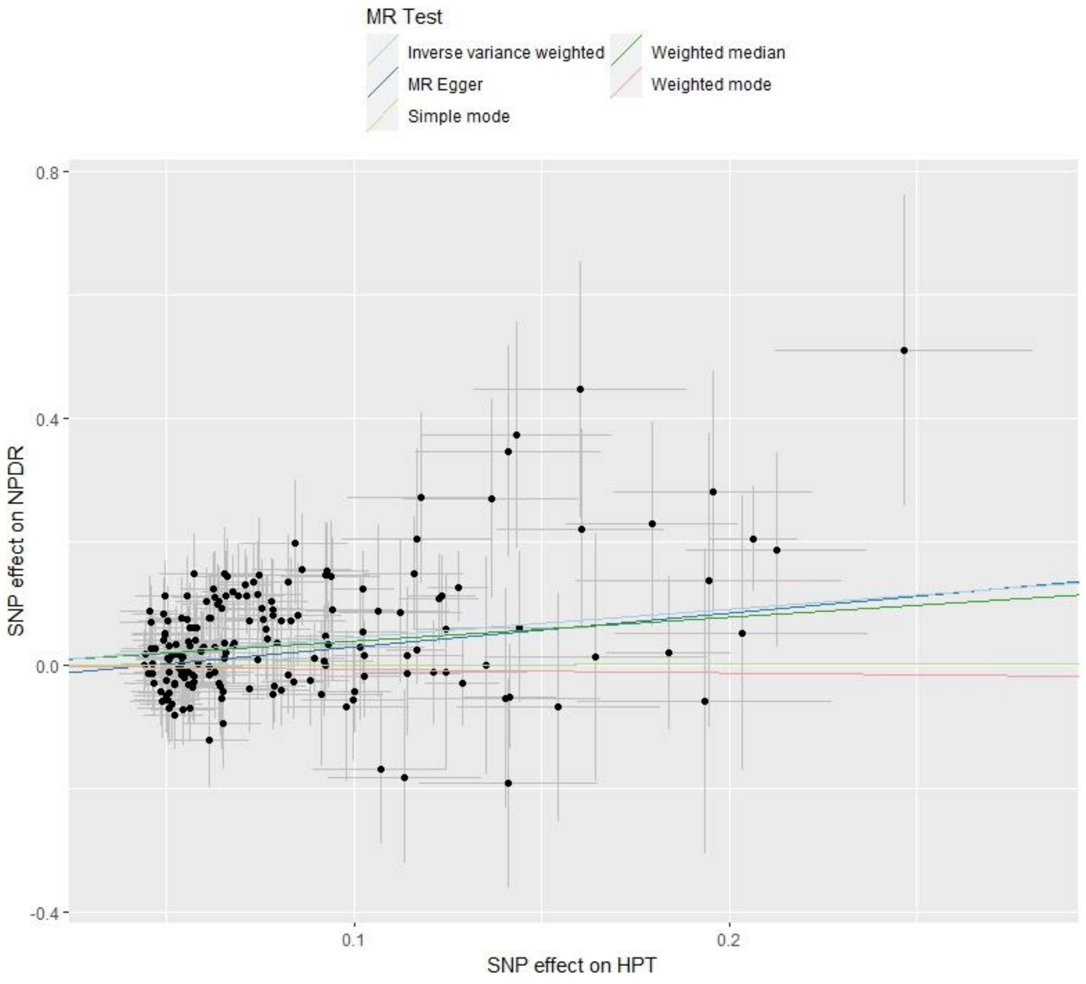

Supplemental Figure 8D. Scatter plots of the MR analysis: HPT-PDR.

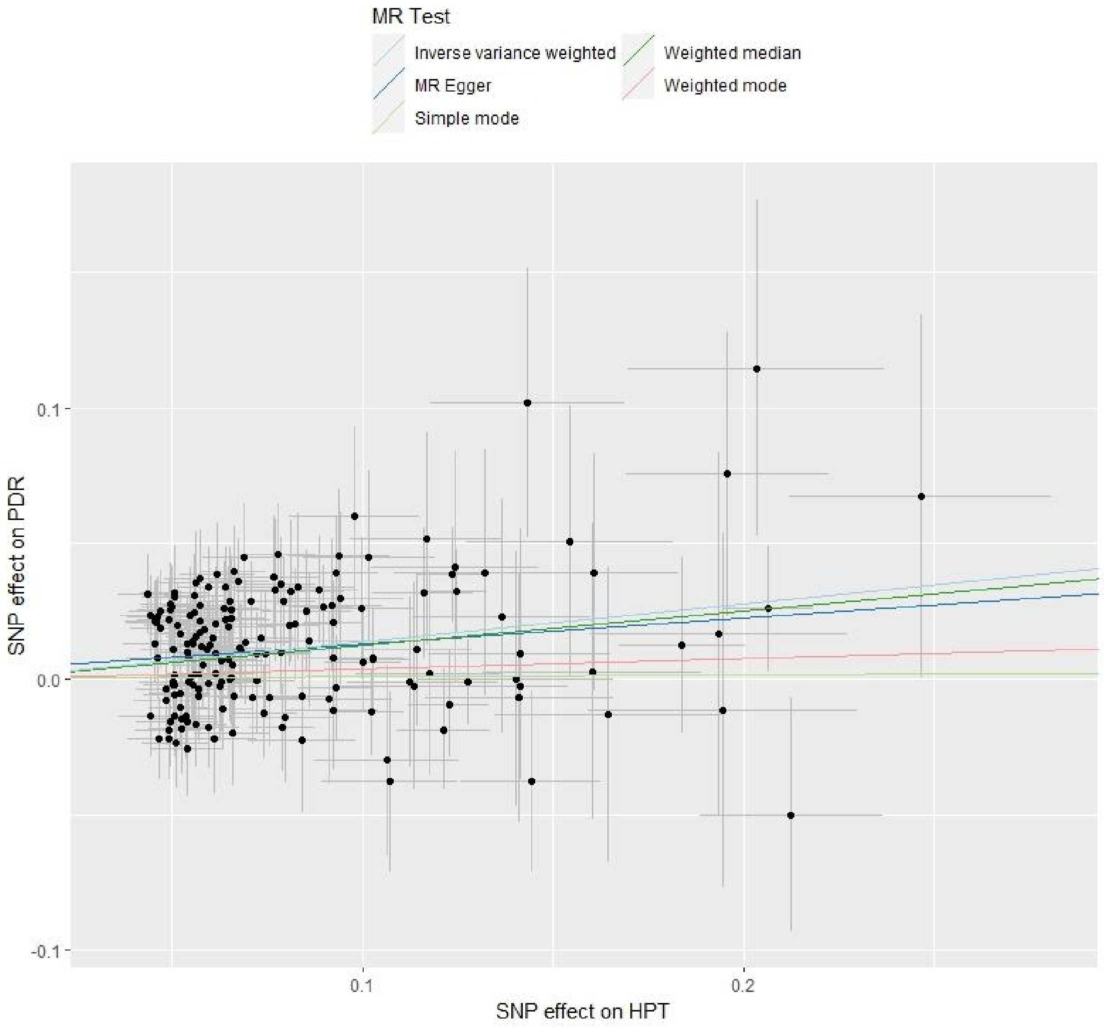

Supplemental Figure 9A. Scatter plots of the MR analysis: GD-DBR.

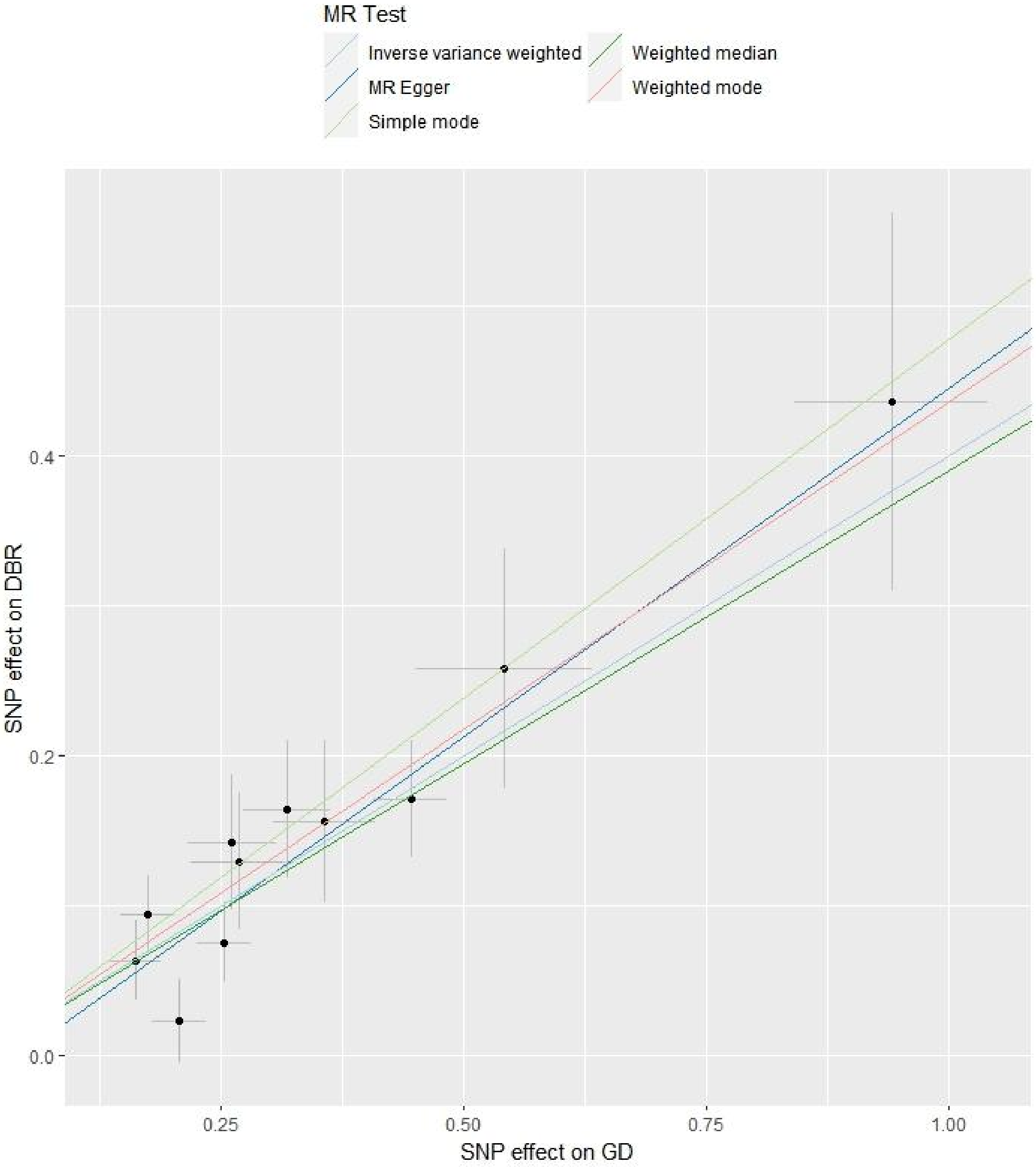

Supplemental Figure 9B. Scatter plots of the MR analysis: GD-DR.

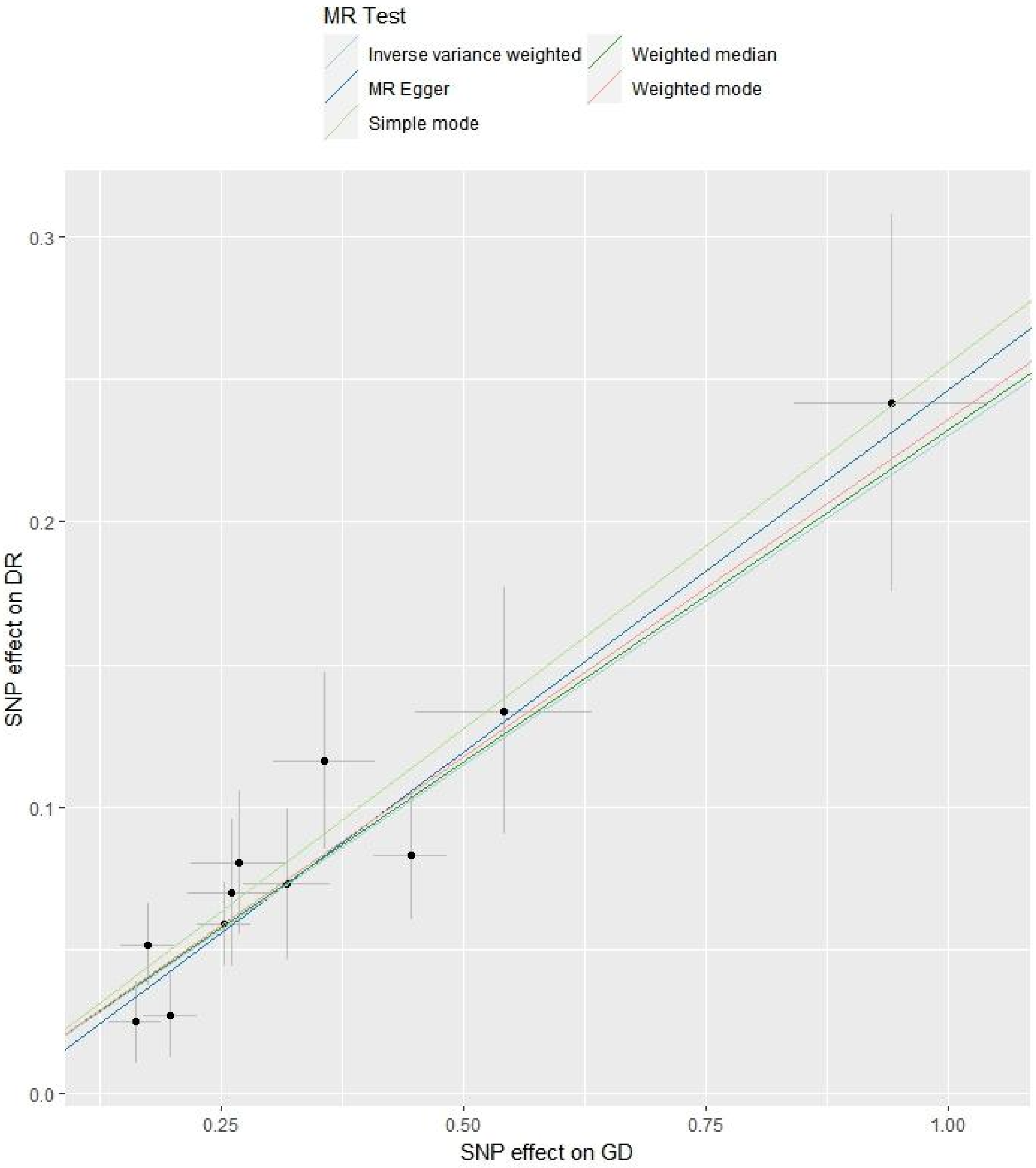

Supplemental Figure 9C. Scatter plots of the MR analysis: GD-NPDR.

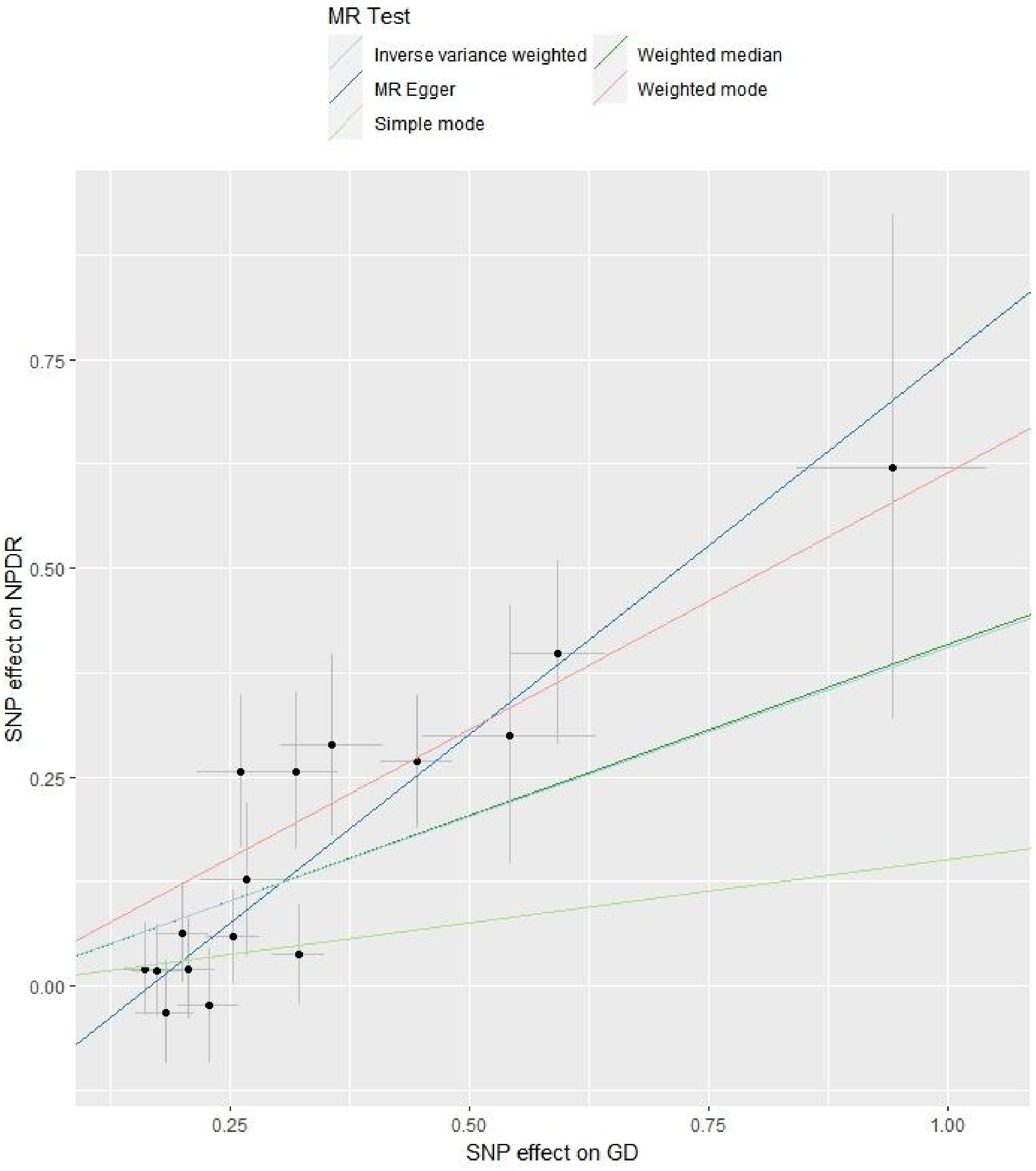

Supplemental Figure 9D. Scatter plots of the MR analysis: GD-PDR.

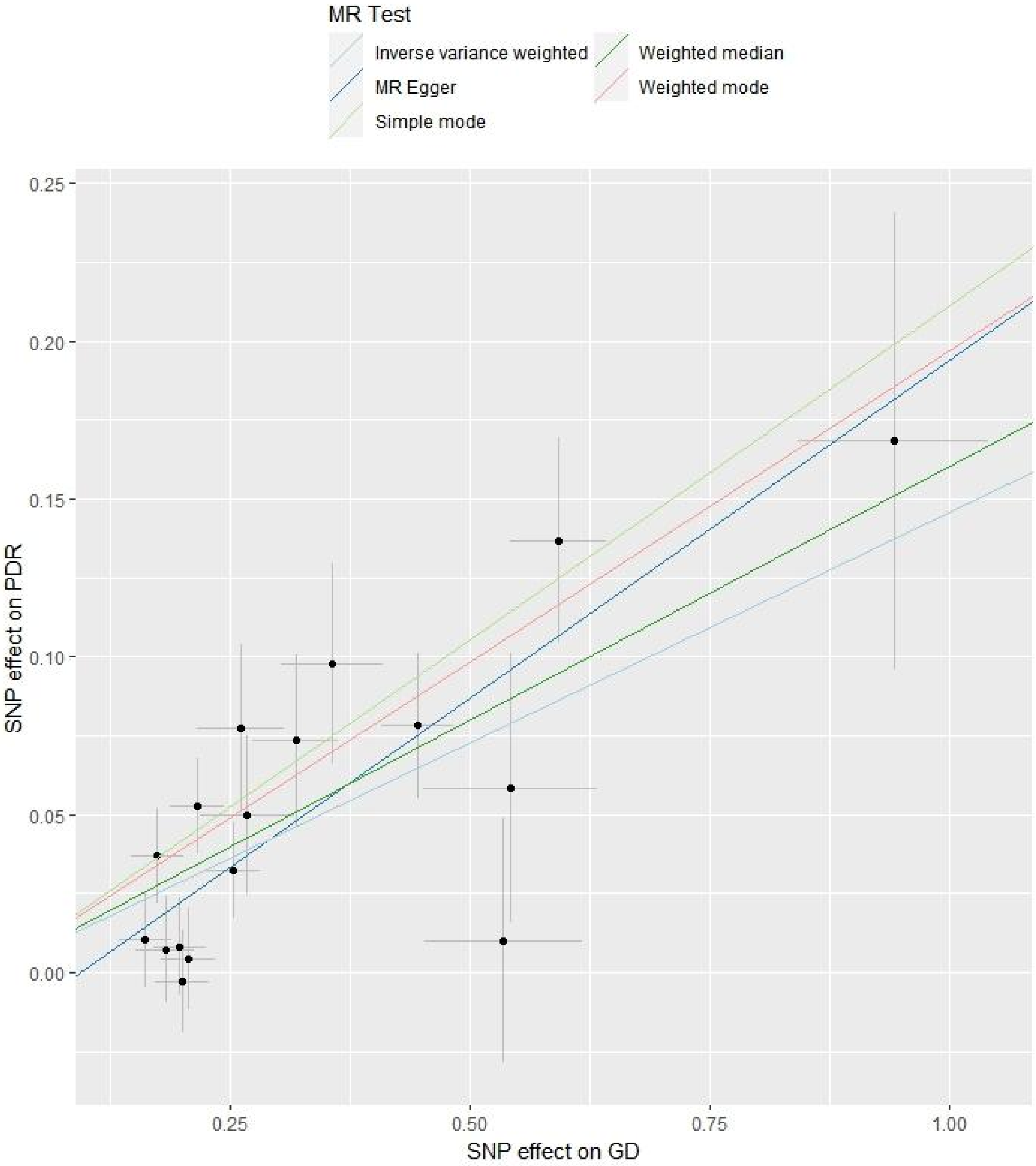

Supplement: Supplementary file 2 [file Image1.pdf]
